# The NADPH Oxidase Complexes in *Botrytis cinerea*: Evidence for a Close Association with the ER and the Tetraspanin Pls1

**DOI:** 10.1371/journal.pone.0055879

**Published:** 2013-02-13

**Authors:** Ulrike Siegmund, Jens Heller, Jan A. L. van Kann, Paul Tudzynski

**Affiliations:** 1 Institut fuer Biologie und Biotechnologie der Pflanzen, Westfaelische Wilhelms Universitaet Muenster, Muenster, Germany; 2 Laboratory of Phytopathology, Wageningen University, Wageningen, The Netherlands; Maastricht University, The Netherlands

## Abstract

NADPH oxidases (Nox) are major enzymatic systems that generate reactive-oxygen species (ROS) in multicellular eukaryotes. In several fungi they have been shown to be involved in sexual differentiation and pathogenicity. However, in contrast to the well characterized mammalian systems, basic information on the composition, recruitment, and localization of fungal Nox complexes and on the molecular mechanisms of their cellular effects are still lacking. Here we give a detailed analysis of components of the Nox complexes in the gray mold fungus *Botrytis cinerea.* It had previously been shown that the two catalytic transmembrane subunits BcNoxA and B are important for development of sclerotia and for full virulence, with BcNoxA being involved in spreading of lesions and BcNoxB in penetration; BcNoxR functions as a regulator of both subunits. Here we present evidence (using for the first time a functional GFP fusion able to complement the ΔbcnoxA mutant) that BcNoxA localizes mainly to the ER and at the plasma membrane; BcNoxB shows a similar localization pattern, while the regulator BcNoxR is found in vesicles throughout the hyphae and at the hyphal tip. To identify possible interaction partners, which could be involved in the localization or recruitment of the Nox complexes, we functionally characterized the tetraspanin Pls1, a transmembrane protein, which had been suggested to be a NoxB-interacting partner in the saprophyte *Podospora anserina*. Knock-out experiments and GFP fusions substantiate a link between BcNoxB and BcPls1 because both deletion mutants have overlapping phenotypes (especially a defect in penetration), and the proteins show a similar localization pattern (ER). However, in contrast to the corresponding protein in *P. anserina* BcPls1 is important for female fertility, but not for ascospore germination.

## Introduction

Reactive oxygen species (ROS) play a dual role in eukaryotic cells: as unwanted by-products of many metabolic reactions they have to be scavenged to avoid deleterious effects on macromolecules, but their diffusible nature enables them to play a role as messenger molecules for signaling within the cell and in cell-cell communication [1]. Multiple roles of ROS are also evident in host-pathogen interactions. Plants react to pathogen attack with the so-called oxidative burst, which generates ROS serving primarily as signaling molecules for induction of effective plant defense reactions like the hypersensitive response (HR). In addition ROS can have direct toxic effects on the invading pathogen. On the other hand, ROS are involved in differentiation processes of fungal pathogens, which are important for virulence [2]. The necrotrophic gray mold fungus *Botrytis cinerea* initiates a massive oxidative burst upon infection of its host plants and even requires the host plant’s response to achieve full pathogenicity: in HR-deficient *Arabidopsis thaliana* strains the virulence of *B. cinerea* is strongly reduced [3]. *B. cinerea* - like many other fungi - was shown to produce ROS within and around hyphal tips also *in planta* [4]. Therefore, it was postulated that the fungus uses ROS to actively trigger the host response [5], [6]. Searching for the source of this extracellular ROS we initiated a functional characterization of the major enzymatic ROS-generating system in eukaryotic cells, the NADPH oxidase complex, in *B. cinerea*.

NADPH oxidases are transmembrane proteins which reduce oxygen to superoxide using NADPH as an electron donor. The best studied member of this group is the mammalian phagocytic gp91^phox^ (named also Nox2), which is responsible for the defense response of neutrophils [7]. To activate its function a multi-subunit complex has to be formed: the integral membrane protein flavocytochrome b_558_, consisting of the catalytic subunit gp91^phox^ and the adaptor protein p22^phox^ [8], is assembled with the cytosolic components p40^phox^, p47^phox^, p67^phox^ and the small GTPase Rac upon phosphorylation of p47^phox^ [9]. NADPH oxidases have been found in a wide range of eukaryotic organisms, and have originally been linked with multicellularity [10]. However, recently a genuine NADPH oxidase was identified in *Saccharomyces cerevisiae* [11] showing that this correlation is no longer valid. In filamentous fungi three distinct subfamilies of Nox enzymes have been discovered: NoxA, NoxB and NoxC [12], [13]. NoxA and NoxB are homologs of the mammalian catalytic subunit Nox2 and are widely distributed within filamentous fungi. The third isoform NoxC, present in some species, carries a calcium binding EF-hand, like the human Nox5 and the plant Nox homologs (Rbohs) [14]. Furthermore, in filamentous fungi homologs of the mammalian regulatory subunit p67^phox^ (NoxR) and the small GTPase Rac are present [15], [16]. Other components of the fungal Nox complexes are yet to be discovered, as neither homologs of the mammalian cytosolic subunits p47^phox^ and p40^phox^ nor the flavocytochrome component p22^phox^ have been identified in fungi [16]. Possible components might be the scaffold protein Bem1 and the guanine exchange factor (GEF) Cdc24, as a direct interaction of these proteins with the putative regulator NoxR via their PB1 domains was shown in *Epichloe festucae* [17]. Functional analyses of Nox genes in filamentous fungi have revealed that Nox are involved in various differentiation processes. This was first demonstrated in *Aspergillus nidulans*, where NoxA is required for fruiting body formation [12]. Subsequently, it was shown that also in the ascomycetes *Podospora anserina* and *Neurospora crassa* Nox1 is necessary for development of fruiting bodies. The second Nox enzyme present in *P. anserina* and *N. crassa* (Nox2) is involved in germination of ascospores [18], [19]. In *B. cinerea* both BcNoxA and B are involved in sclerotial formation [20]. Additionally, it was shown recently that in *P. anserina* both Nox complexes are required for cellulose degradation via specialized needle-like hyphal structures [21]. Besides their involvement in sexual differentiation processes, Nox are also needed during pathogenicity. In *B. cinerea* deletion mutants of *bcnoxA* penetrate the host tissue, but colonization of the host is slower compared to the wild-type (WT), and *bcnoxB* mutants are slower in forming primary lesions. The deletion of the regulator *bcnoxR* shows an additive effect [20]. In *Claviceps purpurea* the deletion mutant of *cpnox1* is impaired in colonization of host tissue: it produces conidia-containing honeydew and immature sclerotia only in rare cases [22]. In contrast *cpnox2* deletion results in significantly increased production of honeydew. However, the mutants are unable to form mature sclerotia and are therefore restricted to the sporulating stage (D. Buttermann, pers. communication). In the endophyte *Epichloe festucae* deletion of *efnoxA* leads to a switch from a mutualistic to an antagonistic interaction, while deletion of *efnoxB* has no effect on the plant-interaction phenotype [23]. In *Magnaporthe oryzae* deletion of both *nox1* and *nox2* caused apathogenicity of the fungus [24]. Interestingly, the effect of *nox* deletion on ROS production varies between the different species. Some show unchanged cellular ROS levels (*B. cinerea*, *M. oryzae* single mutants), in some cases they are enhanced (*M. oryzae* vegetative hyphae of Δnox1Δnox2, *P. anserina* Δnox1, Δnox1/2 and ΔnoxR) [20], [21], [24], and in some cases they are reduced (*A. nidulans* ΔnoxA during sexual development; *E. festucae* ΔnoxA; *M. oryzae* Δnox1/2 during appressoria formation) [12], [24], [25].

Given that the catalytic subunits NoxA and NoxB are transmembrane proteins, it is still not known to which cellular membranes they localize. So far the only localization study was accomplished by Egan *et al*., who showed Nox1 localization in *M. oryzae* at the appressorium periphery starting from 4 h and in the central appressorium vacuole after 24 h [24]. This *gfp-*construct was under control of the native promoter, but was not shown by complementation studies to be functional.

As the constitution of the fungal Nox complex is still incompletely elucidated, possible partners for direct or indirect interaction are of high interest. In *P. anserina* the Nox complex was recently linked to another transmembrane protein, the tetraspanin Pls1, because the deletion of *pls1* and *nox2* led to a similar germination defect in ascospores [26]. Tetraspanins are small eukaryotic integral membrane proteins [27] known to have varying functions. In the animal kingdom they function as organizers of multimolecular membrane complexes and regulate, amongst others, cell migration, fusion and signaling events [28]. In filamentous fungi three families of tetraspanins have been identified (Pls1, Tsp2, Tsp3) with different distribution amongst phyla [29]. All these tetraspanin proteins display low sequence similarities, but they share highly conserved secondary structures. These include four transmembrane domains and a cysteine-based pattern in the large extracellular loop EC2 [30]. The most important tetraspanin of ascomycetes seems to be Pls1, which was first identified as a virulence factor in *M. oryzae*. Expression of *pls1* was shown in appressoria, where the protein localizes to the plasma membrane and in vacuoles [31]. As shown for *M. oryzae*, Pls1 is also necessary for appressoria-mediated penetration in *Colletotrichum lindemuthianum* and the *B. cinerea* strain T4 [32], [33]. Interestingly, *pls1* and *nox2* seem to have a similar distribution within fungal genomes, as they are either both present or both absent, suggesting a connection between these genes or their products, respectively [26].

Here we present evidence that the two NADPH oxidase catalytic subunits BcNoxA and BcNoxB localize in the ER and at the plasma membrane, while their regulator NoxR localizes in vesicles throughout the hyphae and at the hyphal tip. Furthermore, the tetraspanin Pls1 is functionally characterized in the more aggressive *B. cinerea* strain B05.10 to substantiate a possible connection between NoxB and Pls1. Indeed, both deletion mutants have overlapping phenotypes and the proteins show a similar localization pattern.

## Results

In a previous study NADPH oxidase components *bcnoxA* (BC1G_10823.1) and *bcnoxB* (BC1G_14597.1), encoding the catalytic subunits, and *bcnoxR* (BC1G_06200.1), encoding the regulatory subunit, were identified in *B. cinerea* B05.10. Functional characterization of the respective deletion mutants revealed an involvement of all components in pathogenicity and sclerotia production [20]. Even though the composition and possible up- and downstream partners of the Nox complex are intensely investigated in several fungi, so far many components remain to be elucidated. As Lambou *et al*. [26] recently proposed a connection between the tetraspanin Pls1 and the catalytic NADPH oxidase subunit NoxB, we were interested in the characterization of the tetraspanin Pls1 in *B. cinerea* B05.10 to investigate a possible connection to the BcNox complex.

### Identification of the Gene *bcpls1* in *B. cinerea* B05.10

A blastx search in the *B. cinerea* B05.10 database (Broad Institute, http://www.broadinstitute.org/annotation/genome/botrytis_cinerea/Home.html) using the *Magnaporthe oryzae* Pls1 sequence as a query identified an MgPls1 homolog protein annotated as hypothetical protein similar to tetraspanin (BC1G_09439.1). According to the annotation, the open reading frame (ORF) BC1G_09439.1 consists of 612 bp and encodes a protein of 176 aa. However, 88 bp upstream of the annotated start codon, there was no sequence information in the B05.10 sequence. Comparison of the genomic B05.10 DNA sequence with expressed sequence tags (ESTs) of the strain B05.10 and the genomic sequence of *B. cinerea* T4 (http://urgi.versailles.inra.fr/Species/Botrytis) indicated that the actual start codon of BC1G_09439.1 is 147 bp upstream of the annotated start codon (data not shown). Therefore the ORF BC1G_09493.1, named *bcpls1*, consists of 759 bp. Comparison of ESTs with *bcpls1* confirmed an intron of 81 bp. Therefore, *bcpls1* encodes a protein of 226 aa. The predicted protein contains no conserved domains. Still, a prediction of transmembrane helices in proteins using the TMHMM server version 2.0 (http://www.cbs.dtu.dk/services/TMHMM) indicated the structural hallmarks of tetraspanins with four transmembrane domains and a large extracellular loop between TM3 and TM4 (data not shown).

### Generation of Δbcpls1 Mutants

The gene *pls1* had already been characterized in the wild-type strain T4 of *B. cinerea* [33]. However, this strain shows some phenotypical differences to the strain B05.10 (which is widely used as reference strain in the *Botrytis* community): it sporulates in darkness, does not produce any sclerotia or oxalic acid and is less aggressive during infection. In order to analyze the function of BcPls1 in *B. cinerea* B05.10 and compare it to the function of other proteins in this strain, a *bcpls1* deletion strain was created using a replacement approach. The ORF of *bcpls1* in the wild-type strain B05.10 was replaced by a hygromycin resistance cassette by homologous recombination (Fig. S1A). Replacement of *bcpls1* was detected in several transformants by diagnostic PCR. Single-spore isolations resulted in homokaryotic deletion mutants, which were confirmed by diagnostic PCR (Fig. S1B). Southern blot analysis confirmed homologous integration into the *bcpls1* locus without further ectopic integration events of the replacement cassette in three independent transformants, termed Δbcpls1 (T20), Δbcpls1 (T21) and Δbcpls1 (T23) (Fig. S1C). The mutants Δbcpls1 (T20) and Δbcpls1 (T21) showed the same phenotype in all experiments, while the mutant Δbcpls1 (T23) showed a stronger phenotype when grown on minimal medium. Therefore Δbcpls1 (T20) was taken for all further analyses.

### BcPls1 has no Influence on Light-dependent Differentiation but Affects Sexual Development

Differentiation and growth rates of the Δbcpls1 deletion mutant were compared to the wild type (WT) in plate assays. These analyses revealed that light-dependent differentiation of Δbcpls1 was not disturbed: the mutant produced conidia when grown in light and sclerotia of normal appearance when grown in darkness (Fig. S2A). Furthermore, Δbcpls1 was neither affected in growth on complete and minimal medium nor in growth on media containing different oxidative or osmotic stressors (Fig. S2B). In addition, the Δbcpls1 mutant did not show significantly altered extracellular H_2_O_2_ production, as shown by DAB staining and an Amplex Red peroxide assay (although the latter showed a slight induction of H_2_O_2_ production by the mutant (P<0.05)) or acidification of the surrounding medium as compared to the WT (Fig. S3B). The same analyses were also done for ΔbcnoxA, ΔbcnoxB, ΔbcnoxR and ΔbcnoxAB. All of these *bcnox* mutants showed WT-like production of oxalic acid and H_2_O_2_ production was not reduced in any of them. Indeed the ΔbcnoxA mutant even showed an increased production of H_2_O_2_ (P<0.01). In contrast, treatment of the WT with the flavoenzyme inhibitor diphenyleneiodonium chloride (DPI) in the Amplex Red assay showed a reduction of H_2_O_2_ production of about 50% compared to the untreated WT (P<0.01), indicating that besides the Nox enzymes other flavoenzymes are involved in extracellular H_2_O_2_ production (Fig. S3).

**Figure 1:**
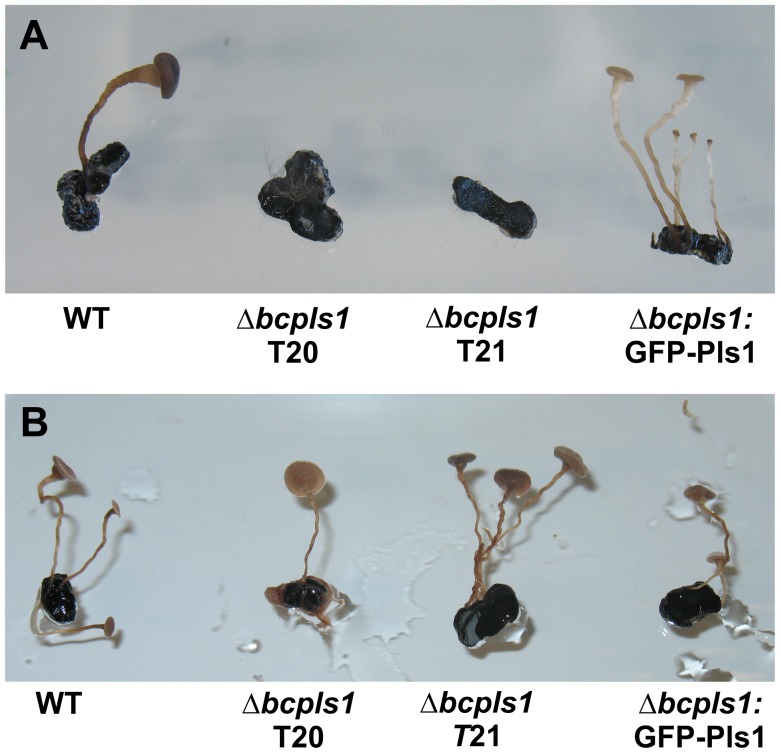
Sexual crosses of the WT and Δbcpls1 mutant. **A:** Sexual crosses between *B. cinerea* SAS405 microconidia (standard strain of the opposite mating type to B05.10) and sclerotia of the B05.10 WT, two independent Δbcpls1 transformants and the Δbcpls1:GFP-Pls1 complementation strain. Sclerotia of the Δbcpls1 deletion strain show no morphological differences during their development but are sterile. **B:** Sexual crosses between *B. cinerea* SAS405 sclerotia and microconidia of the B05.10 WT, the Δbcpls1 mutants and the Δbcpls1:GFP-Pls1 complementation strains. Microconidia of the Δbcpls1 deletion strain are fertile.

However, results of sexual crosses with *B. cinerea* SAS405 (the MAT1-2 reference strain commonly used as opposing mating partner of B05.10) showed an unusual behavior of the Δbcpls1 mutant. Although the Δbcpls1 sclerotia exhibited no morphological differences during their development, they failed to develop apothecia after fertilization with SAS405 microconidia. In contrast, the sclerotia of SAS405 when fertilized with microconidia of the Δbcpls1 mutant produced apothecia of normal appearance (Fig. 1). In conclusion, sclerotia of the Δbcpls1 mutant are non-functional in sexual development, i.e. the mutant is female sterile, while the microconidia of the mutant are functional.

Ascospores were collected from apothecia obtained from the cross between SAS405 sclerotia and Δbcpls1 microconidia and germinated at low density (∼50–100 spores/plate) on non-selective medium. The ascospores showed normal germination rates. Upon transfer to plates containing hygromycin, at least half of the germlings were hygromycin-resistant, indicating that these germlings carried the Δbcpls1 mutation. Therefore, in contrast to *P. anserina* [26], Pls1 is not required for ascospore germination in *B. cinerea*. We previously showed that BcNoxA, BcNoxB and BcNoxR are involved in production of sclerotia. Deletion of *bcnoxA* and *bcnoxR* led to complete loss of sclerotia, whilst deletion of *bcnoxB* resulted in the formation of fewer and morphologically aberrant sclerotia. However, ascospores derived from sexual crosses between microconidia of these deletion mutants and SAS405 sclerotia resulted in normal apothecia and the ascospores developing from these showed normal germination rates [20].

### BcPls1 is not Involved in Germination of Asexual Conidia or Vegetative Hyphal Fusion Events

Recently it was shown that *B. cinerea*, as many other fungi, forms conidial anastomosis tubes (CATs) resulting in vegetative hyphal fusions. In this process the BcNox complex is involved: while the deletion of *bcnoxB* leads to reduced hyphal fusion rates, deletion of *bcnoxA* and *bcnoxR* leads to complete loss of germling fusions [34]. To compare BcPls1 and the subunits of the BcNox complexes the ability of Δbcpls1 to form CATs was analyzed.

**Figure 2:**
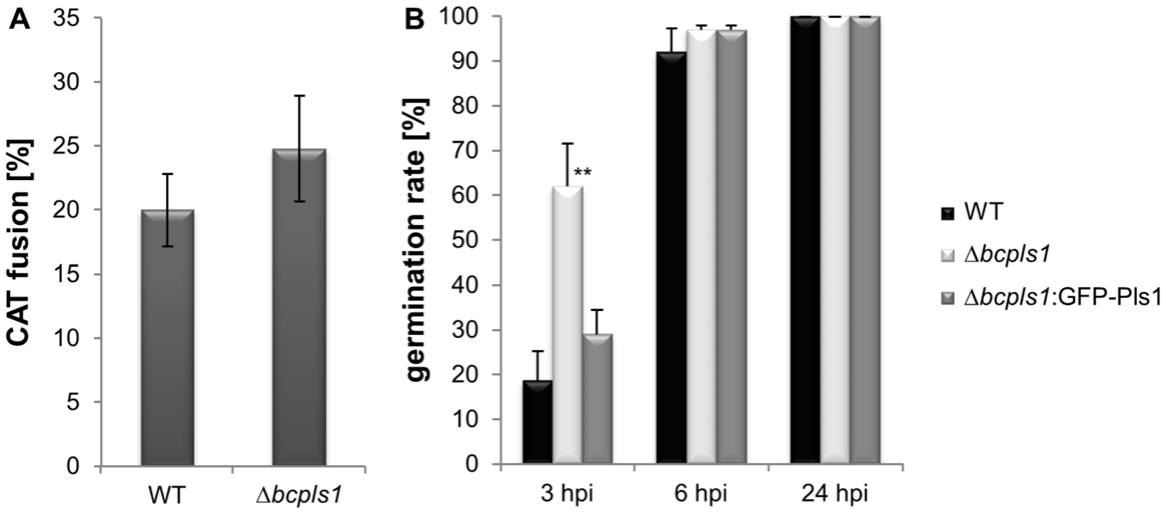
Germination assay and vegetative hyphal fusions of Δbcpls1 and the WT. A: Hyphal germling fusions of the WT and the Δbcpls1 mutant. 3×10^7^ conidia were plated on Vogel’s minimal medium and incubated for 14 h in darkness. CAT formation was analyzed using light microscopy. The diagram represents the mean values of three replicates evaluating 300 spores each. The fusion frequency (CAT fusion %) was slightly enhanced in the **Δ**bcpls1 mutant; statistical analysis (T-test) revealed no significant differences. **B:** Comparison of germination efficiencies of *B. cinerea* B05.10 WT and Δbcpls1 conidia after 3, 6 and 24 hours. Germination was monitored in liquid Gamborg-B5 medium amended with 10 mM glucose. The diagram represents one experiment with triplicate counting. Germination rates of **Δ**bcpls1 conidia were increased at 3 hpi. Asterisks above the bars denote significant differences in the measurements of the indicated strains to the WT. ** = P<0.01.

Interestingly, the fusion frequency of Δbcpls1 germlings was slightly enhanced when compared to the WT (Fig. 2A). However, 3 h after inoculation also the sugar-induced germination of asexual conidia was significantly increased in the deletion mutant. Hence, the increased germination rate might account for the more frequent fusion events observed in the Δbcpls1 mutant (Fig. 2B). Therefore, in summary, regarding the CAT formation the Δbcpls1 phenotype is clearly distinct from the ΔbcnoxA phenotype, and closer to the ΔbcnoxB phenotype, which is still able to form CATs [34].

### BcPls1 has an Impact on Pathogenicity

**Figure 3:**
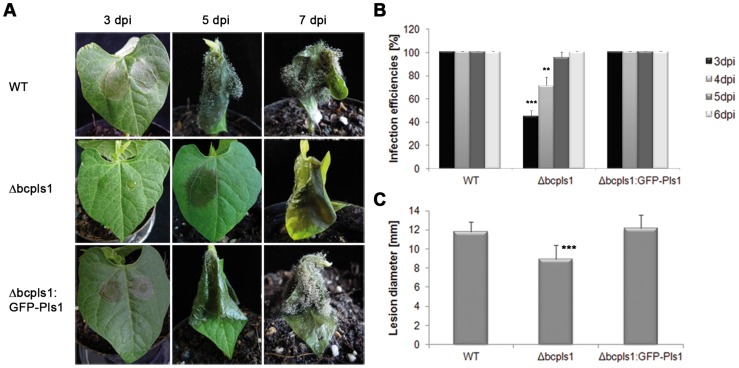
Virulence of the *Botrtytis cinerea* strain Δbcpls1 in comparison to the wild-type B05.10 (WT) and the complemented strain Δbcpls1:GFP-Pls1. **A:** Time course of infection of French bean (*Phaseolus vulgaris*). Primary leaves of 10 day old plants were inoculated with 7 µl droplets of conidial suspensions (2×10^5^ spores/ml in Gamborgs B5+2% glucose). Progression of infections was documented 3, 5 and 7 days after the infection (dpi). Results of the mutant were not consistent as in some cases infection could be detected between 3 and 5 dpi (see middle row and B). However, at 7 dpi all the leaves infected with the mutant strain showed strong infection symptoms (see right row). **B:** Statistical evaluation of infection efficiencies at different time points. At 3 dpi in only about 50% of the Δbcpls1-infected leaves symptoms could be detected while infection efficiencies of the WT and the complemented strain amounted to 100%. At 6 dpi also in the Δbcpls1 strain infection symptoms were detected in 100% of infected leaves ((separate set of experiments from A: repeated 3 times with a sample size of n = 16, 16, 12 per strain; standard deviations are indicated by the error bars). **C:** Statistical evaluation of infection spreading. Only the lesion diameters of successful infection events were measured at 3 dpi. Lesion diameters of Δbcpls1 infections were only slightly reduced when compared to WT and to the complemented strain at this early time point (The indicated values are means of 16 different infection events; standard deviations are indicated by the error bars; a biological replicate yielded similar results). Asterisks above the bars denote significant differences in the measurements of the indicated strains to the WT. ** = P<0.01; *** = P<0.001.

The main defect of the ΔbcnoxB mutant is its performance in the early stages of pathogenicity [20]. According to these findings BcPls1 might also be required for infection in the strain B05.10. Therefore, the virulence of Δbcpls1 was compared to the WT in a pathogenicity test (Fig. 3A). This test showed that indeed the virulence of the *bcpls1* deletion mutant was reduced. However, the B05.10-derived mutant was not completely apathogenic, as reported for the T4-derived mutant [33]. In fact, the lesion diameter of the B05.10-derived mutant at 3 dpi was only moderately reduced compared to controls, when considering only the diameters of lesions in successful infections (Fig. 3C). However, by that assessment time only ca. 50% of Δbcpls1 inoculated bean leaves showed any symptoms of infection, while the inoculations of the WT were 100% successful, as demonstrated in a separate set of experiments in Fig. 3B: the infection efficiencies of the mutant gradually increased until at 6 dpi infection was successful in 100%. Whenever there was an infection the mutant was able to complete the whole life cycle nearly as rapidly as in the WT. In conclusion, Δbcpls1 showed reduced penetration efficiency, while the course of infection was normal once the mutant had entered the host. In comparison the infection pattern of ΔbcnoxB showed delayed formation of primary lesions in all cases (1 day slower than the WT), but once it entered the host the infection was also normal [20].

### BcPls1, BcNoxB and BcNoxR are Required for Penetration

**Figure 4:**
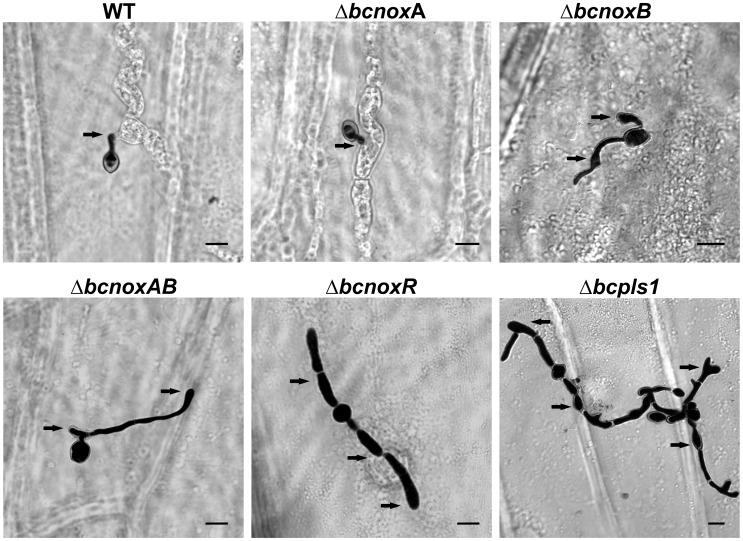
Penetration assay on onion epidermis. The hydrophobic side of onion epidermis was inoculated with 10 µl droplets of conidial suspensions (5×10^4^ spores/ml). After 24 h incubation at 18°C lactophenole blue staining was used to visualize the fungus on top of the onion epidermis. Fungal cells within the onion cells are not stained. Conidia of the WT and ΔbcnoxA form a short germ tube with a terminal thickening, which are able to directly penetrate the plant surface. Conidia of the deletion strains ΔbcnoxB, ΔbcnoxR, ΔbcnoxAB and Δbcpls1 germinate on the plant epidermis and differentiate appressoria-like structures, but fail to penetrate the plant via these structures. Black arrows indicate appressoria-like structures. Scale bars = 10 µm.

**Figure 5:**
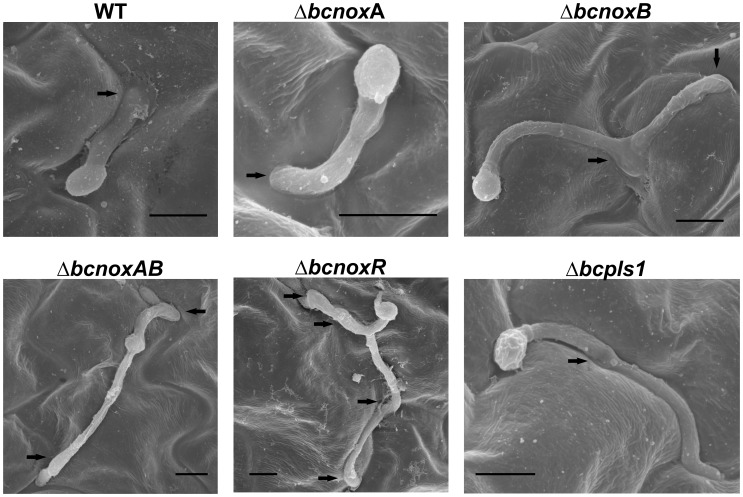
*In planta* penetration assay on bean leaves. Scanning electron microscopy (SEM) images of germinating conidia of the *B. cinerea* WT, Δbcpls1, ΔbcnoxA, ΔbcnoxB, ΔbcnoxAB and ΔbcnoxR were taken on the surface of bean leaves. Detached bean leaves were inoculated with conidial suspensions (2×10^6^ spores/ml) and 18 h after the infection samples were prepared for SEM by glutharaldehyde fixation, osmification, ethanol series, critical point drying and gold sputtering. Arrowheads indicate appressoria-like structures. Scale bars = 10 µm.

As pathogenicity assays revealed that BcNoxB, BcNoxR and BcPls1 seem to be necessary for penetration in *B. cinerea* B05.10, the ability of the Δbcpls1 deletion mutant to penetrate onion epidermal strips was investigated. The deletion mutants of the *bcnox* genes as well as the double mutant of the catalytic subunits BcNoxA and BcNoxB (ΔbcnoxA, ΔbcnoxB, ΔbcnoxAB, ΔbcnoxR) were included for comparison. To exclude the possible impact of host defense reactions affecting the assay, onion epidermises were inactivated prior to inoculation with conidial suspensions and the penetration ability of the strains was studied microscopically 24 hpi (Fig. 4). These analyses showed that Δbcpls1 still produced swollen hyphal tips (appressoria-like structures) resembling those produced by the WT, but whereas the WT successfully penetrated the epidermis via these structures, the mutant was incapable of doing so. A similar phenotype has been observed for the ΔbcnoxB mutant in prior studies and was confirmed again here. Comparable phenotypes were also observed for ΔbcnoxAB and ΔbcnoxR, while the ΔbcnoxA mutant was able to penetrate the onion epidermis 24 hpi [20]. On bean leaves a similar effect was expected. To analyze penetration ability in this system leaves of bean plants were inoculated with conidial suspensions of the WT, the Δbcpls1 mutant and the Δbcnox mutants; 18 hpi the samples were prepared for analysis via scanning electron microscopy (SEM) (Fig. 5). This analysis revealed that in most cases the WT as well as ΔbcnoxA produced short germ tubes before penetration of the host tissue. In contrast, the majority of germ tubes observed for ΔbcnoxB, ΔbcnoxAB, ΔbcnoxR and Δbcpls1 elongated on the surface of the epidermis of the host leaf and instead of penetrating the host by appressoria-like structures the mutants continually produced new hyphal outgrowths and cycles of appressoria-like structures. This phenotype has previously been described for the ΔbcnoxR mutant [2]. The observation that Δbcpls1, ΔbcnoxB and ΔbcnoxR mutants all show a similar defect in the penetration via appressoria-like structures suggests a connection between BcPls1 and the Nox complex.

### BcNoxA, BcNoxB and BcPls1, but not BcNoxR, are Localized in the ER

**Figure 6:**
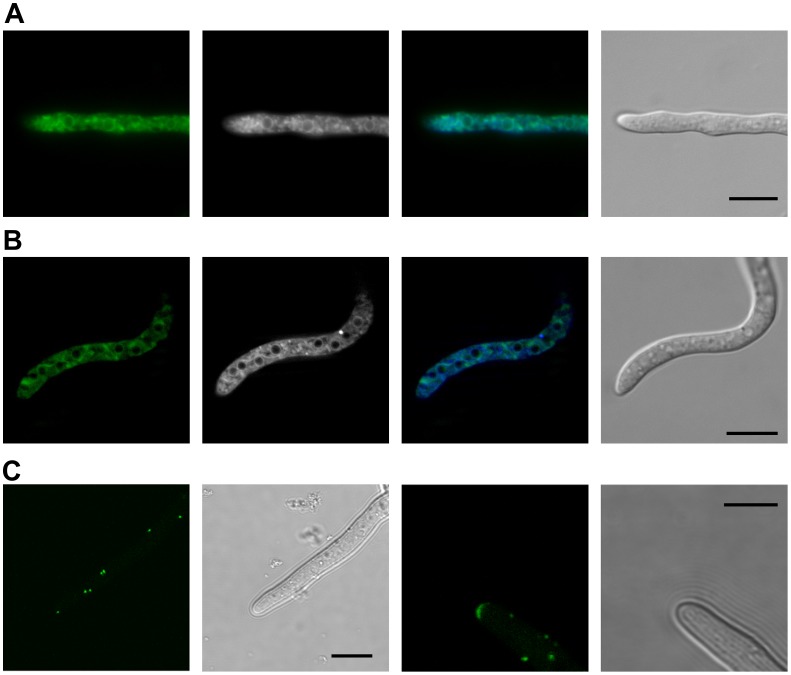
Cellular localization of BcNoxA, BcNoxB and BcNoxR. Protein localization was determined by epifluorescence microscopy in germlings of the strains WT:GFP-NoxA, WT:NoxB-GFP and WT:GFP-NoxR expressing *gfp-bcnoxA, bcnoxB-gfp* and *gfp-noxR* gene fusions, respectively. 10-µl droplets of a conidial suspension in GB5 medium (10^5^ conida/ml) were placed on a glass slide and allowed to germinate over night before microscopic analyses were performed. **A**: BcNoxA localized to intracellular membrane structures and at times also to the plasma membrane. Staining with the ER-Tracker™ Blue-White DPX and a respective overlay show that the intracellular structures are partly consistent with the ER (from left to right: GFP-NoxA, ER-tracker, overlay, white light). **B**: BcNoxB localized to similar intracellular membrane structures and to the plasma membrane as visible for BcNoxA. Staining with the ER-Tracker™ Blue-White DPX and a respective overlay show that the intracellular structures visual are similar to ER structures (from left to right: NoxB-GFP, ER-tracker, overlay, white light). **C**: BcNoxR accumulated in cellular granules, which were distributed irregularly throughout the hyphae (left) and at the hyphal tip, where it seemed to determine the point of outgrowth (right, see also movie S2). Scale bars = 10 µm.

In mammalian cells Nox have been shown to fulfill various functions in a range of tissues, therefore localization of the Nox isoforms varies considerably in mammals. In fungi only three isoforms have been identified and in *B. cinerea* only two catalytic subunits (BcNoxA and BcNoxB) and their putative regulator (BcNoxR) were found. Differing functions have been ascribed to BcNoxA and BcNoxB. Therefore, the question arises as to whether their distinct functions are based on different localizations. In order to localize the proteins N-terminal (BcNoxA) or C-terminal (BcNoxB) *gfp*-fusions were generated and introduced into *B. cinerea* B05.10 (B05.10:GFP-NoxA, B05.10:NoxB-GFP). The constructs were controlled by the constitutive *oliC* promoter. Conidia of strains containing NoxB:GFP and GFP:NoxA, respectively, were allowed to germinate on a glass slide and the germlings were then examined by epifluorescence microscopy (Fig. 6A and B). Surprisingly, despite differing functions, BcNoxA and BcNoxB both localize to similar structures. The strongest fluorescence was visible for dynamic, and to some extent spherical, structures within the hyphae. To identify these structures germlings were stained with ER-Tracker™ Blue-White DPX. Co-localization of the ER-tracker and GFP-NoxA showed partial overlap, with accentuated localization of NoxA around the nuclei. For NoxB-GFP colocalization with the ER-tracker showed largely consistent signals (Fig. 6A and B). Additionally germlings were stained with Hoechst 33342. The fluorochromic property of this dye is attributed to its ability to bind with DNA structures, but it can also stain cell walls and septae, depending on the pH [35]. Hoechst staining showed that the structures visible seemed to enclose the nuclei (Fig. S5). A similar localization pattern has been described for the endoplasmatic reticulum (ER) membrane system in connection with the outer membrane of the nuclear envelope [36]. In addition, faint fluorescence was at times visible in both strains at the plasma membrane surrounding the hyphae (Fig. S5).

Due to its homology to the mammalian Nox-regulator subunit p67^phox^ NoxR is thought to be necessary for regulation of NoxA and NoxB [16]. Furthermore, it was previously shown that NoxR is connected to NoxA and NoxB activation [15], [18], [20]. Therefore we studied the localization of the regulator BcNoxR by introducing a GFP-BcNoxR fusion construct in *B. cinerea* B05.10. Germinated conidia were examined as described above for this construct and it strikingly showed a quite distinct localization from the previously observed localization of BcNoxA and BcNoxB (Fig. 6C). BcNoxR most prominently localized to cellular granules distributed irregularly throughout the hyphae. These granules seemed to move randomly within the hyphae (Brownian motion), however, time lapse showed that the granules had the tendency to stay within the young parts of the hyphae, close to the hyphal tips (see movie S1). In addition, in growing hyphae BcNoxR localized also to hyphal tips, where it seemed to be associated with the origin of outgrowth and the growth direction (Fig. 6C). In some of these hyphae additional material coming from the granules was transported to the growing hyphal tip (see movie S2).

**Figure 7:**
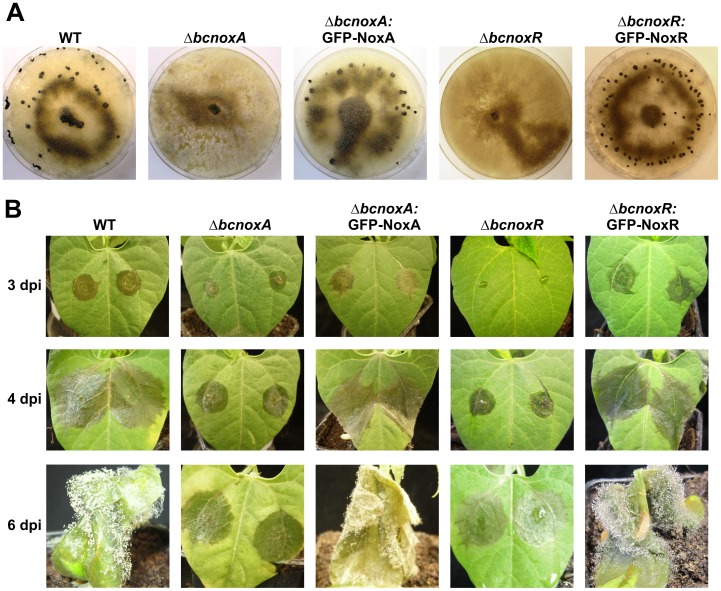
Complementation analyses of ΔbcnoxA and bcnoxR with *gfp*-constructs. Strains ΔbcnoxA:GFP-NoxA and ΔbcnoxR:GFP-NoxR expressing *gfp-noxA* and *gfp-noxR* gene fusions were tested for their phenotypical characteristics. The strains WT, ΔbcnoxA and ΔbcnoxR served as a control. The GFP-fusion constructs did complement the deletion phenotypes of ΔbcnoxA and ΔbcnoxR **A**: The ability to form sclerotia after 2 weeks incubation in darkness on CM media at 18°C is restored. **B**: The pathogenicity defect of the mutants is reinstated. Primary leaves of 10 day old French bean plants were inoculated with 7 µl droplets of conidial suspensions (2×10^4^ spores/ml in Gamborgs B5+2% glucose). Progression of infections was documented 3, 4 and 6 days after the infection (dpi).

In order to make sure that the used *gfp*-fusion constructs are functional, complementation studies were performed. The constructs GFP-NoxA and GFP-NoxR showed similar localization patterns in the deletion mutants as the respective construct in the WT (Fig. S4) and complemented their phenotypes (Fig. 7). No transformants were obtained with the BcNoxB-GFP construct in ΔbcnoxB background.

**Figure 8:**
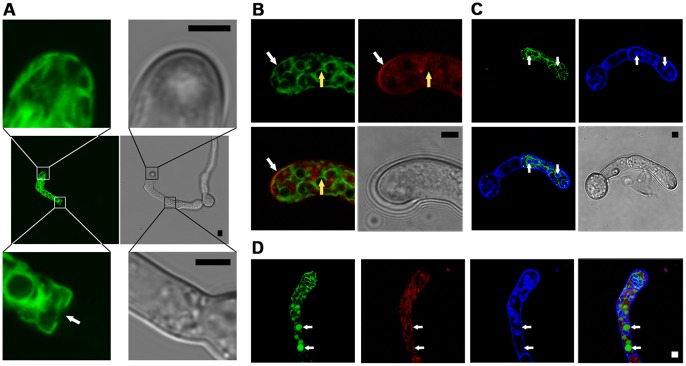
Cellular localization of the protein BcPls1. Protein localization was determined by confocal laser scanning microscopy (CLSM) in germlings of the strain Δbcpls1:gfp-Pls1 expressing a *gfp-bcpls1* gene fusion. 10-µl droplets of a conidial suspension in GB5 medium (10^5^ conida/ml) were given on a glass slide and were allowed to germinate over night before microscopic analyses were performed. **A**: Overview of BcPls1 localization in a *Botrytis cinerea* germling. BcPls1 mainly localizes to the border of circular structures in germinating hyphae that seem to be coherent (middle row). Higher magnification showed that the protein also localizes to structures at the edge of the hyphal tip (top row). Fluorescence is clearly limited to the last segment of the germling. The septal pore shows no fluorescence (white arrow, bottom row). **B**: Germlings were treated with the endocytosis marker FM4-64 (red, top right panel) to stain intracellular membranes. BcPls1 localization is shown in green (top left panel). White arrows indicate co-localization of BcPls1 fluorescence and the FM4-64 stained plasma membrane (see overlay, bottom left panel). Yellow arrows indicate localization of BcPls1 in membrane structures that are not stained by FM4-64 (indicative for the nuclear envelope/endoplasmic reticulum). **C**: Germlings were treated with the dye Hoechst (blue, top right panel) to stain nuclei. BcPls1 localization is shown in green (top left panel). White arrows indicate Hoechst- stained nuclei that are surrounded by the intracellular membrane structures BcPls1 localizes to ((see overlay, bottom left panel). **D:** Germlings were treated with the dyes FM4-64 (red, second row)) and Hoechst (blue, third row), simultaneously. BcPls1 localization is shown in green (first row). In addition to the membrane structures seen before (nuclear envelope), white arrows indicate localization of BcPls1 to the interior space of round structures, whose membranes are stained by FM4-64 (indicative for vacuoles). Scale bars in all images = 3 µm.

Since in *Botrytis* the deletion mutants Δbcpls1, ΔbcnoxB and ΔbcnoxR show similar penetration defects on onion epidermis and bean leaves, respectively, we also performed localization studies with BcPls1. Therefore an N-terminal fusion of *bcpls1* and *gfp* was transformed into the B05.10 WT and the Δbcpls1 deletion mutant (B05.10:GFP-BcPls1, Δbcpls1:GFP-BcPls1). The construct was controlled by its native promoter and hence showed its native expression pattern. Accordingly, the phenotype of the deletion mutant could be restored (Figs. 1 and 3). To observe localization of the protein, the conidia were treated as described above and analyzed microscopically. These analyses revealed that BcPls1 localized to similar intracellular membrane structures (presumably ER and plasma membrane) as BcNoxB and BcNoxA (Fig. 8). However, while BcNoxB/BcNoxA localized to those membranes within the whole germling, BcPls1 was only detected in the last thickened compartments of some hyphae, which might be appressoria-like structures (Fig. 8A, see also movie S3). Nevertheless, localization to similar membrane structures might hint at a spatial connection between the Nox complex and BcPls1 during formation of specific differentiation structures (in this case appressoria-like structures).

In conclusion BcNoxA, BcNoxB and BcPls1 localize to the ER and at times also to the plasma membrane, while strikingly BcNoxR is, under the tested conditions, found in different structures: it localizes to growing hyphal tips and in cellular granules within the hyphae.

## Discussion

Following our first report on the Nox complexes in *B. cinerea* [20], we present here a deeper analysis of the functions of the complex components focusing on the localization of the catalytic NADPH oxidase subunits BcNoxA and BcNoxB and their regulatory subunit BcNoxR. Furthermore, we initiated a detailed analysis and localization studies of the tetraspanin Pls1 of *B. cinerea* in strain B05.10, which had been suggested to be connected to NoxB, due to their associated distribution in fungal genomes and similar phenotypes of the respective deletion mutants in *P. anserina* [26].

**Figure 9:**
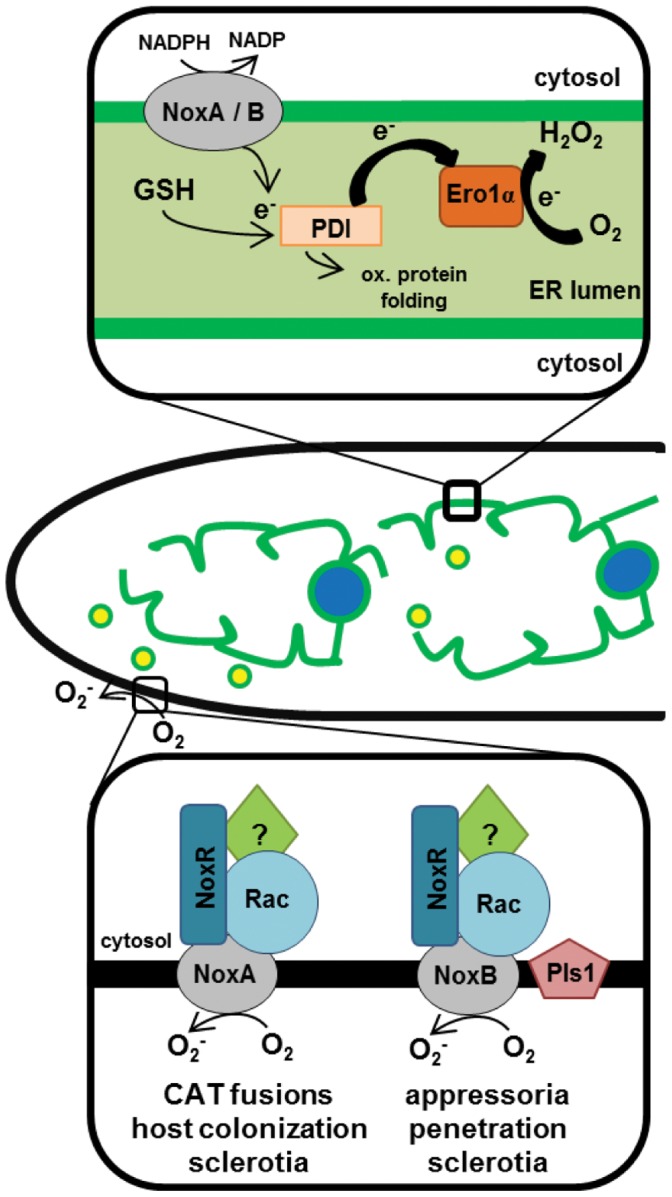
Hypothetical schematic overview of the localization and putative functions of Nox in *B. cinerea*. Nox in *B. cinerea* might function at two different locations. On the one hand (top) they are located in the ER (green) membrane, where they possibly contribute to the ER redox status. Besides GSH (glutathion), Nox might be a second supplier of electrons to PDI. PDI is oxidized by Ero1 by transfer of electrons to O_2_, which in turn becomes H_2_O_2_ (scheme of Ero1-PDI pathway based on [72]. Upon an activation signal Nox are translocated to the plasma membrane (bottom) via vesicles (yellow dots), where they form the active Nox complex with the subunits NoxR, Rac and other so far unknown components (possibly Bem1 and Cdc24 [17]). This complex then transfers electrons through the membrane, reducing oxygen to superoxide, using NADPH as an elctron donor. The BcNoxA complex is necessary for CAT fusions, colonization of plant tissue and sclerotia formation, BcNoxB, which additionally might be regulated by BcPls1, is involved in penetration via appressoria-like structures and female fertility. Nuclei are depicted in blue.

Little is known about the localization of Nox complexes in filamentous fungi: only in *M. oryzae* has Nox1 been shown directly to localize at the appressorium during pathogenic development. We showed here that the catalytic subunits BcNoxA and BcNoxB in *B. cinerea* both localize to the nuclear envelope, the ER and at times to the plasma membrane, whereat BcNoxA showed more concentrated localization around the nuclei, while BcNoxB localization was more dominant within the ER. Since Nox are transmembrane proteins postulated to be located in the outer membrane and to produce extracellular ROS, an ER localization of the complex was not assumed. However, until now there is no clear evidence for extracellular ROS derived from Nox. During pathogenic development extracellular ROS could either be used directly to attack the host, or they could serve as messenger molecules that are perceived to activate internal signaling processes. Interestingly, in the *bcnox* deletion mutants (and in Δbcpls1) decreased ROS levels were neither detected in axenic culture by DAB staining or use of Amplex Red nor *in planta* using NBT staining [20]. In fact for ΔbcnoxA and Δbcpls1 even a moderate enhancement was detected, which was also previously reported for other fungi [20], [21], [24]. The observed reduction of H_2_O_2_ production after DPI treatment substantiates the unspecific effect of this inhibitor and supports the now generally accepted idea that secreted ROS in fungi is not produced by Nox but by alternative flavoenzymes. Hence, in *B. cinerea* a contribution of the Nox to extracellular ROS production is doubtful, and accordingly, the Nox might localize to a compartment other than the outer membrane. Even though we show a localization of BcNoxA and possibly BcNoxB in the ER, both proteins do not possess any ER retention signals. However, retention of a protein in the ER can result from various factors like the lack of a sorting signal, the presence of a signal for retrieval from pre-Golgi compartments or properties of the transmembrane domain (TMD) [37]. The exact mechanism of ER retention remains to be elucidated [38]–[40]. Integral predictions of protein locations (ProtComp v. 9.0) were done resulting in a prediction of all analyzed Nox proteins (*S. sclerotiorum*, *M. oryzae, E. festucae, N. crassa, A. nidulans, P. anserina, B. cinerea, Trichoderma reseei, Homo sapiens*) at the plasma membrane (see Table S1), except Nox2 from *T. reseei* which is predicted at the ER. The fact that the *H. sapiens* Nox1, 2 and 4 have repeatedly been shown to localize and function at the ER [41]–[42] shows that software based localization predictions may differ from real localization. Recently, the yeast Nox Yno1 was also shown to be present in the ER [11] like mammalian Nox1, Nox2 and Nox4 [41], [42]. Besides a primary localization in the ER [43], Nox4 was reported to translocate to the plasma membrane when complexed with the stabilizing component p22^phox^ [44], revealing that localization and function of a protein might not be restricted to a specific compartment or that proteins can be located in the ER for a period of time until they are needed and released. For example the chitin synthase 2 (Chs2) is expressed in the metaphase and retained in the ER through phosphorylation until further mechanisms stop this phosphorylation and release Chs2 from the ER.[45]–[48]. According to these findings it is difficult to decide whether the ER is the final location of the catalytic BcNox subunits, or whether they are stored and modified there in order to be translocated. The fact that the GFP-BcNoxA fusion construct complemented the phenotype of the deletion mutant proves the functionality of the construct and supports a correct localization. However, the function of BcNoxA and BcNoxB within the ER is still unclear. A link between the ER and Nox might be the protein disulfide isomerase (PDI), which is a dithiol–disulfide oxidoreductase chaperone. In mammalian systems Nox1, Nox2, Nox4 and p22^phox^ were linked to PDI [49], [50]. PDI carries out different activities, amongst others it serves as a protein-folding catalyst for intramolecular relocation of disulfide bonds [51], [52]. The main interactor of PDI is the ER flavoprotein Ero1 (ER oxidoreductin 1) [53]. Together, PDI and Ero1 play an essential role in oxidative protein folding [54] and they integrate ER function to cellular redox homeostasis [51]. Therefore the superoxide producing Nox might be involved in maintaining the redox level within the ER in connection with PDI (see also Fig. 9).

In contrast to the catalytic subunits, their regulator BcNoxR localized to cellular granules and the hyphal tip. An interaction, and therefore a similar localization, of NoxR and NoxA/B seems probable as they are homologs to the mammalian Nox2 and p67^phox^ subunits, which come together to form the active complex [16]. Nevertheless, attempts to show a direct interaction between NoxR and NoxA or B in fungi have so far been unsuccessful. BiFC and yeast two-hybrid analysis (based on the split-ubiquitin system) couldn’t proof this interaction in *B. cinerea* so far (U. Siegmund and P. Tudzynski, unpublished data). It has to be taken into account that the formation of the active NADPH oxidase complex is a strictly regulated mechanism, which – in fungi – has not been completely elucidated. The interaction, and therefore similar localization, may only take place under specific conditions in the cell, when the active complex is needed. Thus BcNoxR might be stored in cellular compartments (the visible granules) and upon Nox activation translocates towards the ER-membrane where it interacts with the catalytic subunits. Alternatively, the catalytic subunits BcNoxA and B could have BcNoxR-independent roles, as shown for Nox4 in mammals [43] (see also Fig. 9).

Another possible role for Nox worth investigating in the future could be their involvement in cytoskeleton organization. This was previously postulated for the mammalian system [55] and the recently identified yeast Nox (Yno1) was shown to have an impact on the cell cycle and possibly influences actin assembly [11].

Clearly not all the components of fungal Nox complexes have been identified yet. The previously postulated link between NoxB and Pls1 (similar phenotypes and linked presence in fungal genomes [26]) encouraged us to investigate this connection. Here, we show that as in *B. cinerea* T4 BcPls1 is also necessary for appressoria-mediated penetration in the more aggressive strain B05.10. However, in contrast to Pls1 in T4, lack of BcPls1 in B05.10 does not lead to apathogenicity. The infection rate is reduced, but not completely blocked, and once the fungus has entered the host, infection is comparable to the WT. Preliminary expression data supports an involvement of BcPls1 in formation of appressoria (6–24 hpi), as *bcpls1* is primarily expressed at 12 and 24 hpi (J. Heller and J. van Kan, unpublished data). This fits to the localization studies presented here indicating the presence of BcPls1 in the ER and plasma membrane of hyphae only in the last (apical) segment, especially in hyphae forming appressoria-like structures (see also movie S3). The infection structures in *B. cinerea* had been termed “appressoria-like” because they lack the typical closed septum allowing the generation of turgor pressure and other strictly localized physiological reactions [56]. The results presented here indicate that the whole final hyphal compartment constitutes a separated physiological unit, sealed by the first septum. The precise role of Pls1 remains to be elucidated. Besides the impediment during appressoria formation, differentiation and growth of the *bcpls1* mutant appears normal. Interestingly, Δbcpls1 forms sclerotia in WT-like manner, but crossing experiments showed that these sclerotia are sterile. This is an uncommon phenotype, because most female sterile mutants in *B. cinerea* thus far described, with the exception of the Δbcfrp1 mutant, fail to form any sclerotia [57].

There are several overlapping phenotypes of ΔbcnoxB and Δbcpls1 supporting the assumed functional link of both proteins, the most obvious one being the penetration defect. However, there are slight differences: while in the *bcnoxB* mutant primary lesion formation is generally retarded [20], Δbcpls1 is able to penetrate in 50% of the cases 3 dpi like the WT, in the other cases primary lesion formation is also delayed. Nevertheless, both mutants once they have entered the host are able to colonize tissue like the WT. More detailed analyses of the penetration 18–24 hpi on onion epidermis and French bean leaves revealed a similar defect of Δbcpls1 and ΔbcnoxB resulting in appressoria formation and repeated outgrowth instead of penetration as observed for the WT. In contrast, the ΔbcnoxA mutant penetrates the host like the WT. Another observation supporting the functional link of BcNoxB and BcPls1 is the ability of the mutants to form hyphal fusions via CATs. Deletion mutants of *bcnoxA, bcnoxR* and the double mutant *bcnoxA/B* do not form any hyphal fusions, while ΔbcnoxB is able to form fusions (though with reduced frequency) [34]. Nonetheless, the fact that Δbcpls1 forms WT-like hyphal fusions again relates BcPls1 to BcNoxB rather than to BcNoxA. BcNox and BcPls1 have also corresponding characteristics regarding their control of female fertility. Δbcpls1 produces normal looking sclerotia, but crossing experiments revealed that these sclerotia are sterile. As the *bcnox* mutants are all impaired in sclerotial development (although ΔbcnoxB still produces smaller sclerotia) they are also regarded as female sterile [20]. A final link between BcNox and BcPls1 is their similar localization in the ER and the plasma membrane, although for BcPls1 this localization is only found in the last (apical) segment of the hyphae. This could suggest that the BcNox complexes are involved in different mechanisms controlled by differential regulation systems and that BcPls1 is involved in the activation of BcNoxB e.g. during early stages of infection.

Taken together we have shown that the catalytical Nox subunits BcNoxA and BcNoxB as well as the tetraspanin Pls1 are localized in the ER and the plasma membrane. A distinct localization of the regulator BcNoxR at the hyphal tip and in cellular granules suggests translocation of the cytosolic subunits to the catalytical subunit (NoxA/B) only when the active Nox complex is needed. A better understanding of the activation and assembly mechanisms of the fungal Nox complexes in more detail will be in the focus of further investigations.

## Materials and Methods

### Fungal Strains

*Botrytis cinerea* [teleomorph *Botryotinia fuckeliana* (de Bary) Whetzel] strain B05.10 is a putative haploid derivate of SAS56, an isolate obtained from *Vitis vinifera* after benomyl treatment [58]. This strain is used as a host strain for gene replacement experiments and localization studies, and as a wild-type control in all experiments. All other strains used in this study are listed in Table 1.

**Table 1:** Strains used in this study.

Strain	Genotype	Reference
*B. cinerea* SAS405	MAT1-2	[^58^]
ΔbcnoxA	B05.10 ΔbcnoxA::nat1	[^20^]
B05.10:gfp-noxA	B05.10::gfp-noxA hph	this study
ΔbcnoxA:gfp-noxA	ΔbcnoxA::gfp-noxA hph	this study
ΔbcnoxB	B05.10 ΔbcnoxB::hph	[^20^]
B05.10:noxB-gfp	B05.10::noxB-gfp nat1	this study
ΔbcnoxR	B05.10 ΔbcnoxR::nat1	[^20^]
B05.10:gfp-noxR	B05.10::gfp-noxR hph	this study
ΔbcnoxR:gfp-noxR	ΔbcnoxR::gfp-noxR hph	this study
ΔbcnoxAB	B05.10 ΔbcnoxA::nat1 ΔbcnoxB::hph	[^20^]
Δbcpls1	B05.10 Δbcpls1::hph	this study
Δbcpls1:gfp-pls1	Δbcpls1::gfp-pls1 nat1	this study

### Media and Culture Conditions

Yeast cells were either cultivated in complex media (YPD) (0.5% (w/v) yeast extract, 2% (w/v) glucose, 2% (w/v) peptone, pH 5.8) or for selection in selective drop-out (SD) medium (20 g/l glucose, 6.7 g/l Difco Yeast Nitrogen Base w/o amino acids [BD, Franklin Lakes, NJ USA], 0.77 g/l DO-supplement [Clontech, Mountain View, CA, USA], pH 5.8) at 30°C.

*E. coli* cells were grown in lysogeny broth (LB) medium (10 g/l peptone, 5 g/l yeast extract, 10 g/l NaCl) [59] at 37°C.

WT and mutant strains were grown on different complex media. Potato dextrose agar (PDA) (Sigma-Aldrich Chemie, Steinheim, Germany) was supplemented with 100 g/l homogenized leaves of French beans (PDAB) (*Phaseolus vulgaris*). Synthetic complete medium (CM) was set according to Pontecorvo *et al.* [60]. Growth behavior was also tested on Czapek Dox minimal medium (CD) (20 g/l sucrose, 3 g/l NaNO_3_, 1 g/l K_2_HPO_4_, 0.5 g/l KCl, 0.01 g/l FeSO_4_×7 H_2_O, 0.5 g/l MgSO_4_×7 H_2_O, pH 5.2). In order to obtain conidia, strains were incubated for 6–8 days at 18°C under light conditions (12 h light, 12 h darkness); for sclerotial production, strains were grown 3 weeks at 18°C in darkness. For DNA preparations, mycelium was grown 3–4 days at 18°C on CM agar over-layed with Cellophane. Stress sensitivity tests were performed by inoculating strains on CM plates supplemented with H_2_O_2_, menadione, sorbitol and NaCl.

### Transformation

A knock-out construct for *bcpls1* was obtained using the homologous recombination system in yeast, as described previously [61]. The 5′- and 3′-regions of *bcpls1* (1178 bp and 724 bp) were amplified from genomic DNA using the primer pairs 7/8 and 9/10 (Table S2). These primers not only contain sequences to amplify the 5′- and 3′-regions of the corresponding genes, but also the sequences homologous to the resistance cassette and the yeast plasmid pRS426 [62]. The hygromycin resistance cassette containing the *hph* gene of *E. coli* under control of the *trpC* promoter of *A. nidulans* was generated with primers 11/12 using pCSN44 as a template. The three PCR fragments, together with the *Eco*RI/*Xho*I linearized vector pRS426, were co-transformed into the *Saccharomyces cerevisiae* strain FY834 [63], where homologous recombination took place. Transformants were selected on SD plates lacking uracil. Total DNA from uracil-prototrophic yeast colonies was isolated with the GeneJET™ Plasmid Miniprep Kit (Fermentas), including an initial step to disrupt yeast cells by addition of glass beads. The DNA was then used as template to amplify the *bcpls1* replacement construct by use of primers *7/10* (Table S2). The complete replacement fragment was used to transform *B. cinerea* strain B05.10.

For construction of the *gfp*-*bcpls1* complementation construct, a nourseothricin resistance cassette had to be integrated into the existing vector pBHT2-GFP-BcPls1 (Mathieu Gourgues and Marc-Henri Lebrun, *unpublished data*). The nourseothricin resistance cassette from the vector pEHN1nat1 was amplified using the primers 13/14 and cloned into the vector pSTBlue1 for sequence confirmation. From the vector pSTBlue1 the resistance cassette was excised using the restriction enzyme *Eco*RI and cloned into the *Eco*RI-digested pBHT2-GFP-BcPls1. The resulting vector was linearized using the restriction enzymes *Pvu*I, *Sal*I and *Sph*I and transformed into strain Δbcpls1.

For construction of the *gfp-bcnoxR, gfp-noxA* and *bcnoxB-gfp* fusion constructs the homologous recombination system in yeast was also used [61]. The genes *bcnoxR, bcnoxA* and *bcnoxB* were amplified using the primers 15/16, 19/20 and 17/18, respectively. These primers contain sequences homologous to the glucanase terminator and the *gfp* of the vector pNAH-OGG (*bcnoxR, bcnoxA*) and to the *oliC* promoter and *gfp* of the vector pNAN-OGG (*bcnoxB*) [64]. These vectors include the *bcniiA*-flanks to replace the nitrite reductase-encoding gene of *B. cinerea*, ensuring a defined integration site. Deletion of this gene has no effect on *B. cinerea*, provided nitrogen sources other than nitrate are available [64]. The PCR products were co-transformed into the uracil-auxotrophic *S. cerevisiae* strain FY834 with pNAH-OGG linearized with *Not*I or pNAN-OGG linearized with *Nco*I, respectively, as described above. Plasmidic DNA from uracil-prototrophic yeast colonies was isolated using the SpeedPrep Yeast plasmid isolation kit (DualsystemsBiotech, Switzerland) and transformed into *E. coli*. Single plasmids were isolated, and after sequencing the expression cassette was excised using the enzymes *Apa*I/*Sac*II (bcnoxA, bcnoxR plasmids) and *Apa*I (bcnoxB plasmid) and transformed into strain B05.10.

For transformation of *B. cinerea*, protoplasts were generated using a mixture of glucanex (Novozymes, Denmark), lysing enzyme (Sigma-Aldrich, St Louis, MO, USA) and yatalase (Takara Bio Inc, Shiga, Japan). Protoplasts were transformed according to Schulze-Gronover *et al*. [65] using 300 µl of PCR-product (knock-out) or 25 µg of the linearized vector (GFP- fusions), respectively. Resistant colonies were transferred to agar plates containing GB5 agar, supplemented with 70 µg/ml of hygromycin B (Invitrogen, San Diego, USA) or 70 µg/ml of nourseothricin (Werner-Bioagents, Jena, Germany). Single conidial isolates were obtained by spreading conidial suspensions on GB5 plates containing 70 µg/ml of hygromycin B or 70 µg/ml. The conidia were germinated and single colonies transferred individually to new plates containing the selection marker. Homologous integration of the different transformation constructs was proven by diagnostic PCR (Primers see Table S2) and Southern blots.

### Standard Molecular Methods

Fungal genomic DNA was isolated as described by Cenis [66]. Southern blot analysis was performed according to Sambrook *et al.* [67]. Hybridisation was carried out in 6× SSPE, 5× Denhardts solution, 0.1% SDS, and 50 mM phosphate buffer, pH 6.6, at 65°C for 16 to 20 h in the presence of a random-primed [α-^32^P] dCTP labeled probe. Filters were washed for 10 min in 2× SSC (1× SSC is 0.15 M NaCl plus 0.015 M sodium citrate), 0.1% sodium dodecyl sulfate (SDS) and for 10 min in 1× SSC, 0.1% SDS. Hybridization and washing of the filters were carried out at 65°C.

### Sexual Crosses

Crosses were performed as described by van der Vlugt-Bergmans *et al.* [68]. Cap structures of mature apothecia were collected and placed on a glass microscope slide in a droplet of sterile water. The cap was crushed with a second microscope slide in order to release asci and ascospores. The sample was suspended in 2–3 ml of sterile water and filtered over glass wool to remove large debris. The resulting flow-through contained a suspension of ascospores which was dispersed on appropriate media for further analysis.

### Conidial Germination Tests

For analyses of conidiospore germination on glass surfaces in nutrient dependency [69], conidia were harvested from CM agar plates, filtrated over Nytex and suspended in 10 ml of sterile water and washed three times. A conidial suspension (25 µl) with a concentration of 5×10^5^ spores/ml was placed in the center of a cover slip and 475 µl of B5 nutrient solution were added to the conidia supplemented with glucose at a final concentration of 10.5 mM. The cover slips were incubated in the dark at 20°C as indicated. Germination progress was monitored by light microscopy after 3, 6 and 24 h.

### Vegetative Hyphal Fusion Assay

Vegetative hyphal fusions were tested as described by Roca and Weichert *et al*. [34]. Conidial suspensions were plated on Vogel’s minimal medium [70] and incubated for 14 h. Samples were analyzed using differential interference contrast (DIC) microscopy. For quantification three independent replicates were performed evaluating at least 300 spores each time.

### Pathogenicity Assays

Infection assays were performed with conidia from 10-day-old PDAB-agar cultures. Primary leaves of *Phaseolus vulgaris* L. genotype N90598 (originating from J. D. Kelly, Michigan State University, East Lansing, MI) were inoculated with 7,5 µl of conidial suspensions (2×10^5^ conidia/ml) for the standard pathogenicity test as described by Klimpel *et al*. [71]. The infected plants were incubated in a plastic propagator box at 20°C under natural illumination. Disease symptoms were monitored 2, 3, 4 and 6 days post inoculation (dpi).

### Penetration Assays

For penetration assays on onion epidermal layers, epidermal strips were peeled, washed with distilled H_2_O and incubated at 70°C for 1 hour in a humid chamber (killing). Fungal conidia were harvested, washed three times with double distilled H_2_O and diluted to a concentration of 5×10^4^ spores/ml. 10 µl droplets of this suspension were used for inoculation. The samples were kept in a humid chamber at 18°C for 24 h. For the analysis of penetration ability, samples were analyzed by light microscopy after lactophenol blue staining (staining of extracellular hyphae).

### Statistic Analysis

Independent comparisons of the mutant strains with the B05.10 wild-type strain by two-sample t-tests were performed in Excel (Microsoft).

### Scanning Electron Microscopy (SEM)

For SEM analyses of conidial germination on bean leaves, primary leaves of *P. vulgaris* were inoculated with a 2 × 10^6^/ml conidial suspension in Gamborg’s B5 medium (Duchefa) supplemented with 10 mM glucose and 10 mM KH_2_PO_4_/K_2_HPO_4_, pH 6.4. After 18 h of incubation, 5 mm^2^ pieces of the leaves were excised. The samples were aldehyde fixed, underwent an osmification step and were then dehydrated in an ethanol series, as described by Giesbert et al. (1998). For SEM, samples were critical point dried (Emitech K850 Critical Point dryer; Emitech, Ashford, England), gold sputtered (Emitech vacuum sputter device K550×), and examined with a Hitachi S-3000N scanning electron microscope at 15 kV.

### Confocal Laser Scanning Microscopy (CLSM)

A glass slide was inoculated with 10 µl of a conidial suspension (1×10^5^ conidia/ml) in GB5 medium supplemented with (NH_4_)_2_HPO_4_ (1 mM). Germinated conidia were analyzed microscopically 24 h after inoculation with an inverted microscope (Leica DMIRE2) equipped with a Leica TCS SP2 laser scanning device (Leica Microsystems) using a 63× water-immersion lens. GFP fluorescence was excited using a 488 nm laser line. Images were collected with a resolution of 8 bit using an emission range between 505 nm and 550 nm with a frame average and a line average of 4. For FM4-64 staining a 1∶1000 dilution of FM4-64 (Invitrogen, USA) in GB5 medium was added to the germinating conidia. After a short incubation time FM4-64 fluorescence was excited using a 488 nm laser line. Images were collected with a resolution of 8 bit using an emission range between 700 nm and 750 nm. For Hoechst staining a 1∶1000 dilution of Hoechst 33342 (Frankfurt, Germany) in McIlvaine standard buffer was added to the germinating conidia. After a short incubation time fluorescence was excited using a 405 nm laser line. Images were collected with a resolution of 8 bit using an emission range between 420 nm and 470 nm.

### Epifluorescence Microscopy

A glass slide was inoculated with 10 µl of a conidial suspension (1×10^5^ conidia/ml) in GB5 medium supplemented with 10 mM glucose. Germinated conidia were analyzed 12–24 h after inoculation microscopically with Axio Imager 2 (Zeiss, Jena, Germany) using 63×objective lens or Observer Z.1 (Zeiss, Jena, Germany) using a 20×objective lens.

Hoechst staining was conducted as described for CLSM. For staining of the ER a 1∶50 dilution of ER-Tracker™ Blue-White DPX (Life Technologies, Germany) in Mc Ilvaine standard buffer was added to the germinating conidia. Hoechst staining and the ER-Tracker were examined using the filter set 49 DAPI shift free (excitation G 365, beam splitter FT 395, emission BP 445/50), GFP fluorescence was detected with filter set 38 (excitation BP 470/40, beam splitter FT 495, emission BP 525/50). Images were captured with a Zeiss AxioCam MRm camera and analyzed using the Axiovision Rel 4.8 software package.

### ROS Staining

In order to stain exogenous H_2_O_2_ the strains to be tested were grown on CM plus Cellophane for 3 days. Afterwards, 25 mg of fresh mycelium were over-layed with 1 ml DAB solution (0.5 mg/ml DAB in 100 mM citric acid pH 3.7). The incubation took place for 1.5–2 h in the dark at RT, the evaluation was done visually. Only DAB solution, and DAB solution supplemented with 1 µl H_2_O_2_ (30%) served as a negative controls. As a positive control, 1 µl horseradish peroxidase and 1 µl H_2_O_2_ (30%) were added to the DAB solution.

As an additional assay the production of ROS was measured using Amplex® Red Hydrogen Peroxide/Peroxidase Assay (Life Technologies, Germany). In order to detect H_2_O_2_, 20 mg of semi-dry mycelium were incubated 30 min in a reaction mixture (50 mM sodium phosphate buffer, pH 7.4, 50 µM Amplex® Red reagent and 0.1 U/mL HRP). The in vitro levels of H_2_O_2_ were measured according to the manufacturer’s protocol. Excitation in a range of 530–560 nm and fluorescence emission at 590 nm were detected using the Tecan Safire 96-well microplate reader (Tecan Group Ltd., Switzerland).

## Supporting Information

Figure S1 **Deletion of the gene** ***bcpls1***. **A:** Schematic overview of the deletion strategy. For the detection of homologous integration of the replacement fragments primers were used that bind upstream the 5′-region and within the terminator of the resistance cassette (P3/P4; 5′), or in the promoter of the resistance cassette and downstream the 3′-prime region (P5/P6; 3′), respectively. For detection of purified mutants, primers binding within the wild-type *bcpls1* were used (P1/P2; WT). For Southern blot analyses genomic DNA was digested using the restriction enzyme *Nco*I that cuts within the resistance cassette but not within *bcpls1*. Using the 5′ flank as a probe, in the wild-type a fragment larger than 5 kb was expected and in the mutant a fragment of 3.7 kb. **B:** Diagnostic PCR showing homologous integration of the knock-out fragment at *bcpls1*. **C**: Southern blot showing no further integrations of the knock-out fragment.
(TIF)

Figure S2 **Comparison of growth and differentiation of** ***B. cinerea*** **B05.10 WT and Δbcpls1**. **A:** For analysis of light-dependent differentiation, strains were cultivated on CM for 7 days with a rhythm of 12 h light and 12 h darkness (light) or for 3 weeks in complete darkness (dark). Δbcpls1 shows no differences to the WT. **B:** Plate assays showing growth rates of *B. cinerea* B05.10 and Δbcpls1 on stress causing media. As a control CM was used, oxidative stress was induced using 5 mM H_2_O_2_, 10 mM H_2_O_2_ and 500 mM menadione, osmotic stress was induced using 1 M NaCl and 1 M sorbitol. Colony diameters were measured 3 days after the inoculation. The indicated values are means of five different plates; standard deviations are indicated by the error bars. Asterisks above the bars denote significant differences in the measurements of the indicated strains to the WT. * = P<0.05; ** = P<0.01; *** = P<0.001.
(TIF)

Figure S3 **Production of H_2_O_2_ and oxalic acid in Δbcpls1, ΔbcnoxA, ΔbcnoxB, ΔbcnoxAB, ΔbcnoxR and the WT. A:** Production of H_2_O_2_ was monitored using DAB staining. 25 mg of fresh mycelia were placed in each well to exclude staining differences evolving from differing growth rates of the strains. Strains were previously grown on CM overlayed with cellophane for 3 days. The mycelium was inoculated with DAB solution for 1.5 h in the dark. As negative controls, one well was only filled with DAB solution (-) and one with DAB solution and 1 µl H_2_O_2_ (–). DAB solution, 1 µl H_2_O_2_ and 1 µl horseradish peroxidase served as a positive control (+). **B:** Quantitative analysis of H_2_O_2_ production using Amplex Red Peroxide Assay. 20 mg of fresh mycelia were incubated in the Amplex Red working solution. Strains were previously grown on complete medium (CM) overlayed with cellophane for 3 days, for inhibition of flavoenzymes 100 µM DPI were added to the medium. Fluorescence emission was detected after 30 min at 590 nm (excitation 560 nm). **C:** Acidification of growth medium by oxalic acid production was monitored using the pH indicator bromothymol blue, which was added to the media and turns yellow with decreasing pH value. Plates were inoculated with young mycelium and grown for 3 days. Asterisks above the bars denote significant differences in the measurements of the indicated strains to the WT. * = P<0.05; ** = P<0.01.
(TIF)

Figure S4 **Cellular localization of BcNoxA and BcNoxR in the respective deletion mutants.** Protein localization was determined by epifluorescence microscopy in germlings (in Gamborgs B5+2% glucose) of the strains ΔbcnoxA:GFP-NoxA and ΔbcnoxR:GFP-NoxR expressing *gfp-bcnoxA* and *gfp-noxR* gene fusions, respectively. **A:** BcNoxA localized to intracellular membrane structures and at times also to the plasma membrane. **B:** BcNoxR accumulated in cellular granules, which were distributed irregularly all over the hyphae. Scale bars = 10 µm.
(TIF)

Figure S5 **Localization of BcNoxA and BcNoxB compared with Hoechst-staining of the nuclei.** Protein localization was determined by epifluorescence microscopy in germlings of the strains WT:GFP-NoxA and WT:NoxB-GFP expressing *gfp-bcnoxA* and *bcnoxB-gfp* gene fusions, respectively. 10-µl droplets of a conidial suspension in GB5 medium (10^5^ conida/ml) were placed on a glass slide and allowed to germinate over night before microscopic analyses were performed. **A**: BcNoxA localized to intracellular membrane structures and at times also to the plasma membrane. Hoechst staining and a respective overlay show that the intracellular structures surround the nuclei (from left to right: GFP-NoxA, Hoechst, overlay, white light). **B**: BcNoxB localized to similar intracellular membrane structures and to the plasma membrane as visible for BcNoxA. Hoechst staining and a respective overlay show that the intracellular structures surround the nuclei (from left to right: NoxB-GFP, Hoechst, overlay, white light). Scale bars = 10 µm.
(TIF)

Table S1 **Predicted localization of Nox proteins from various organisms.** Protein sequences of NoxA/1 and NoxB/2 from *B. cinerea, A. nidulans, M. oryzae*, *S. sclerotiorum, E. festucae*, *P. anserina*, *N. crassa* and *Trichoderma reesei* as well as Nox1, Nox2 and Nox4 from *Homo sapiens* were used to predict their cellular localization using ProtComp v. 9.0 (http://linux1.softberry.com/berry.phtml?topic=protcompan&group=programs&subgroup=proloc).
(DOCX)

Table S2 Oligonucleotide primers used in this study.
(DOCX)

Movie S1 **Cellular localization of the protein BcNoxR in growing hyphae.** Protein localization was determined by epifluorescence microscopy in germinating conidia (0–12 hours after inoculation in Gamborgs B5+2% glucose) of the strain B05.10:GFP-BcNoxR expressing a *gfp-bcnoxR* gene fusion. Cellular granules appear in the apical segment of growing hyphae.
(AVI)

Movie S2 **Cellular localization of the protein BcNoxR in growing hyphae.** Protein localization was determined by CLSM analysis in germinating conidia (12 hours after inoculation in Gamborgs B5+2% glucose) of the strain B05.10:GFP-BcNoxR expressing a *gfp-bcnoxR* gene fusion. BcNoxR seems to localize to the hyphal tip. Additional material is added via cellular granules coming from the back of the hyphae.
(AVI)

Movie S3 **Cellular localization of the protein BcPls1 in growing hyphae.** Protein localization was determined by epifluorescence microscopy in germinating conidia (0–14 hours after inoculation in Gamborgs B5+2% glucose) of the strain Δbcpls1:gfp-pls1 expressing a *gfp-bcpls1* gene fusion. GFP fluorescence can be detected from 12 hours in the apical segment of hyphae, showing thickening and growth arrest, which develop into appressoria-like structures.
(AVI)

## References

[1] Wojtaszek P (1997) Oxidative burst: An early plant response to pathogen infection. Biochem J 322 (Pt 3)(Pt 3): 681–692. 10.1042/bj3220681 PMC1218243 9148737

[2] Heller J, Tudzynski P (2011) Reactive oxygen species in phytopathogenic fungi: Signaling, development, and disease. Annu Rev Phytopathol 49: 369–390. 21568704 10.1146/annurev-phyto-072910-095355

[3] Govrin EM, Levine A (2000) The hypersensitive response facilitates plant infection by the necrotrophic pathogen *Botrytis cinerea* . Curr Biol 10(13): 751–757. 10898976 10.1016/s0960-9822(00)00560-1

[4] Schouten A, Tenberge KB, Vermeer J, Stewart J, Wagemakers L, et al (2002) Functional analysis of an extracellular catalase of *Botrytis cinerea* . Mol Plant Pathol 3(4): 227–238. 20569330 10.1046/j.1364-3703.2002.00114.x

[5] Williamson B, Tudzynski B, Tudzynski P, van Kan JAL (2007) *Botrytis cinerea*: The cause of grey mould disease. Molecular Plant Pathology 8(5): 561–580. 20507522 10.1111/j.1364-3703.2007.00417.x

[6] Tudzynski P, Kokkelink L (2009) *Botrytis cinerea*: Molecular aspects of a necrotrophic life style. In: Deising H, editor. Plant Relationships, 2nd Edition The Mycota V. Berlin Heidelberg: Springer-Verlag. 29–50.

[7] Lambeth JD (2004) NOX enzymes and the biology of reactive oxygen. Nature Reviews Immunology 4(3): 181–189. 10.1038/nri1312 15039755

[8] Parkos C, Allen R, Cochrane C, Jesaitis A (1987) Purified cytochrome-B from human granulocyte plasma-membrane is comprised of 2 polypeptides with relative molecular-weights of 91,000 and 22,000. J Clin Invest 80(3): 732–742. 3305576 10.1172/JCI113128 PMC442297

[9] Groemping Y, Lapouge K, Smerdon SJ, Rittinger K (2003) Molecular basis of phosphorylation-induced activation of the NADPH oxidase. Cell 113(3): 343–355. 12732142 10.1016/s0092-8674(03)00314-3

[10] Lalucque H, Silar P (2003) NADPH oxidase: An enzyme for multicellularity? Trends Microbiol 11(1): 9–12. 12526848 10.1016/s0966-842x(02)00007-0

[11] Rinnerthaler M, Buttner S, Laun P, Heeren G, Felder TK, et al (2012) Yno1p/Aim14p, a NADPH-oxidase ortholog, controls extramitochondrial reactive oxygen species generation, apoptosis, and actin cable formation in yeast. Proc Natl Acad Sci U S A 109(22): 8658–8663. 22586098 10.1073/pnas.1201629109 PMC3365156

[12] Lara-Ortiz T, Riveros-Rosas H, Aguirre J (2003) Reactive oxygen species generated by microbial NADPH oxidase NoxA regulate sexual development in *Aspergillus nidulans* . Mol Microbiol 50(4): 1241–1255. 14622412 10.1046/j.1365-2958.2003.03800.x

[13] Aguirre J, Ríos-Momberg M, Hewitt D, Hansberg W (2005) Reactive oxygen species and development in microbial eukaryotes. Trends Microbiol 13(3): 111–118. 15737729 10.1016/j.tim.2005.01.007

[14] Lardy B, Bof M, Aubry L, Paclet MH, Morel F, et al (2005) NADPH oxidase homologs are required for normal cell differentiation and morphogenesis in *Dictyostelium discoideum* . Biochim Biophys Acta 1744(2): 199–212. 15950752 10.1016/j.bbamcr.2005.02.004

[15] Takemoto D, Tanaka A, Scott B (2006) A p67^phox^-like regulator is recruited to control hyphal branching in a fungal-grass mutualistic symbiosis. Plant Cell 18(10): 2807–2821. 17041146 10.1105/tpc.106.046169 PMC1626622

[16] Takemoto D, Tanaka A, Scott B (2007) NADPH oxidases in fungi: Diverse roles of reactive oxygen species in fungal cellular differentiation. Fungal Genet Biol 44(11): 1065–1076. 17560148 10.1016/j.fgb.2007.04.011

[17] Takemoto D, Kamakura S, Saikia S, Becker Y, Wrenn R, et al (2011) Polarity proteins Bem1 and Cdc24 are components of the filamentous fungal NADPH oxidase complex. Proc Natl Acad Sci U S A 108(7): 2861–2866. 21282602 10.1073/pnas.1017309108 PMC3041104

[18] Cano-Dominguez N, Alvarez-Delfin K, Hansberg W, Aguirre J (2008) NADPH oxidases NOX-1 and NOX-2 require the regulatory subunit NOR-1 to control cell differentiation and growth in *Neurospora crassa* . Eukaryotic Cell 7(8): 1352–1361. 18567788 10.1128/EC.00137-08 PMC2519770

[19] Malagnac F, Lalucque H, Lepere G, Silar P (2004) Two NADPH oxidase isoforms are required for sexual reproduction and ascospore germination in the filamentous fungus *Podospora anserina* . Fungal Genet Biol 41(11): 982–997. 15465387 10.1016/j.fgb.2004.07.008

[20] Segmueller N, Kokkelink L, Giesbert S, Odinius D, van Kan JAL, et al (2008) NADPH oxidases are involved in differentiation and pathogenicity in *Botrytis cinerea* . Mol Plant Microbe Interact 21: 808–819. 18624644 10.1094/MPMI-21-6-0808

[21] Brun S, Malagnac F, Bidard F, Lalucque H, Silar P (2009) Functions and regulation of the nox family in the filamentous fungus *Podospora anserina*: A new role in cellulose degradation. Mol Microbiol 74(2): 480–496. 19775249 10.1111/j.1365-2958.2009.06878.x

[22] Giesbert S, Schurg T, Scheele S, Tudzynski P (2008) The NADPH oxidase Cpnox1 is required for full pathogenicity of the ergot fungus *Claviceps purpurea* . Molecular Plant Pathology 9(3): 317–327. 18705873 10.1111/j.1364-3703.2008.00466.x PMC6640299

[23] Tanaka A, Christensen MJ, Takemoto D, Park P, Scott B (2006) Reactive oxygen species play a role in regulating a fungus-perennial ryegrass mutualistic interaction. Plant Cell 18(4): 1052–1066. 16517760 10.1105/tpc.105.039263 PMC1425850

[24] Egan MJ, Wang ZY, Jones MA, Smirnoff N, Talbot NJ (2007) Generation of reactive oxygen species by fungal NADPH oxidases is required for rice blast disease. Proc Natl Acad Sci U S A 104(28): 11772–11777. 17600089 10.1073/pnas.0700574104 PMC1913907

[25] Tanaka A, Takemoto D, Hyon GS, Park P, Scott B (2008) NoxA activation by the small GTPase RacA is required to maintain a mutualistic symbiotic association between *Epichloe festucae* and perennial ryegrass. Mol Microbiol 68(5): 1165–1178. 18399936 10.1111/j.1365-2958.2008.06217.x

[26] Lambou K, Malagnac F, Barbisan C, Tharreau D, Lebrun MH, et al (2008) The crucial role of the Pls1 tetraspanin during ascospore germination in *Podospora anserina* provides an example of the convergent evolution of morphogenetic processes in fungal plant pathogens and saprobes. Eukaryot Cell 7(10): 1809–1818. 18757568 10.1128/EC.00149-08 PMC2568061

[27] Huang S, Yuan S, Dong M, Su J, Yu C, et al (2005) The phylogenetic analysis of tetraspanins projects the evolution of cell-cell interactions from unicellular to multicellular organisms. Genomics 86(6): 674–684. 16242907 10.1016/j.ygeno.2005.08.004

[28] Hemler ME (2005) Tetraspanin functions and associated microdomains. Nat Rev Mol Cell Biol 6(10): 801–811. 16314869 10.1038/nrm1736

[29] Lambou K, Tharreau D, Kohler A, Sirven C, Marguerettaz M, et al (2008) Fungi have three tetraspanin families with distinct functions. BMC Genomics 9: 63. 18241352 10.1186/1471-2164-9-63 PMC2278132

[30] Seigneuret M, Delaguillaumie A, Lagaudriere-Gesbert C, Conjeaud H (2001) Structure of the tetraspanin main extracellular domain. A partially conserved fold with a structurally variable domain insertion. J Biol Chem 276(43): 40055–40064. 11483611 10.1074/jbc.M105557200

[31] Clergeot PH, Gourgues M, Cots J, Laurans F, Latorse MP, et al (2001) Pls1, a gene encoding a tetraspanin-like protein, is required for penetration of rice leaf by the fungal pathogen *Magnaporthe grisea* . Proc Natl Acad Sci U S A 98(12): 6963–6968. 11391010 10.1073/pnas.111132998 PMC34461

[32] Veneault-Fourrey C, Parisot D, Gourgues M, Lauge R, Lebrun MH, et al (2005) The tetraspanin gene ClPls1 is essential for appressorium-mediated penetration of the fungal pathogen *Colletotrichum lindemuthianum* . Fungal Genet Biol 42(4): 306–318. 15749050 10.1016/j.fgb.2005.01.009

[33] Gourgues M, Brunet-Simon A, Lebrun MH, Levis C (2004) The tetraspanin BcPls1 is required for appressorium-mediated penetration of *Botrytis cinerea* into host plant leaves. Mol Microbiol 51(3): 619–629. 14731267 10.1046/j.1365-2958.2003.03866.x

[34] Roca MG, Weichert M, Siegmund U, Tudzynski P, Fleissner A (2012) Germling fusion via conidial anastomosis tubes in the grey mould *Botrytis cinerea* requires NADPH oxidase activity. Fungal Biol 116(3): 379–387. 22385620 10.1016/j.funbio.2011.12.007

[35] Kangatharalingam N, Ferguson MW (1984) A simple and rapid technique for fluorescence staining of fungal nuclei. Curr Microbiol 10(2): 99–103.

[36] Wedlich-Soldner R, Schulz I, Straube A, Steinberg G (2002) Dynein supports motility of endoplasmic reticulum in the fungus *Ustilago maydis* . Mol Biol Cell 13(3): 965–977. 11907275 10.1091/mbc.01-10-0475 PMC99612

[37] Bretscher MS, Munro S (1993) Cholesterol and the golgi-apparatus. Science 261(5126): 1280–1281. 8362242 10.1126/science.8362242

[38] Letourneur F, Cosson P (1998) Targeting to the endoplasmic reticulum in yeast cells by determinants present in transmembrane domains. J Biol Chem 273(50): 33273–33278. 9837899 10.1074/jbc.273.50.33273

[39] Masaki R, Yamamoto A, Tashiro Y (1996) Membrane topology and retention of microsomal aldehyde dehydrogenase in the endoplasmic reticulum. J Biol Chem 271(28): 16939–16944. 8663312 10.1074/jbc.271.28.16939

[40] Kappeler F, Klopfenstein DRC, Foguet M, Paccaud JP, Hauri HP (1997) The recycling of ERGIC-53 in the early secretory pathway - ERGIC-53 carries a cytosolic endoplasmic reticulum exit determinant interacting with COPII. J Biol Chem 272(50): 31801–31808. 9395526 10.1074/jbc.272.50.31801

[41] Ambasta RK, Kumar P, Griendling KK, Schmidt HH, Busse R, et al (2004) Direct interaction of the novel Nox proteins with p22^phox^ is required for the formation of a functionally active NADPH oxidase. J Biol Chem 279(44): 45935–45941. 15322091 10.1074/jbc.M406486200

[42] Petry A, Djordjevic T, Weitnauer M, Kietzmann T, Hess J, et al (2006) NOX2 and NOX4 mediate proliferative response in endothelial cells. Antioxid Redox Signal 8(9–10): 1473–1484. 16987004 10.1089/ars.2006.8.1473

[43] Martyn KD, Frederick LM, von Loehneysen K, Dinauer MC, Knaus UG (2006) Functional analysis of Nox4 reveals unique characteristics compared to other NADPH oxidases. Cell Signal 18(1): 69–82. 15927447 10.1016/j.cellsig.2005.03.023

[44] Von Loehneysen K, Noack D, Jesaitis AJ, Dinauer MC, Knaus UG (2008) Mutational analysis reveals distinct features of the Nox4-p22^phox^ complex. J Biol Chem 283(50): 35273–35282. 18849343 10.1074/jbc.M804200200 PMC2596391

[45] Chuang JS, Schekman RW (1996) Differential trafficking and timed localization of two chitin synthase proteins, Chs2p and Chs3p. J Cell Biol 135(3): 597–610. 8909536 10.1083/jcb.135.3.597 PMC2121060

[46] Zhang G, Kashimshetty R, Ng KE, Tan HB, Yeong FM (2006) Exit from mitosis triggers Chs2p transport from the endoplasmic reticulum to mother-daughter neck via the secretory pathway in budding yeast. J Cell Biol 174(2): 207–220. 16847101 10.1083/jcb.200604094 PMC2064181

[47] Chin CF, Bennett AM, Ma WK, Hall MC, Yeong FM (2012) Dependence of Chs2 ER export on dephosphorylation by cytoplasmic Cdc14 ensures that septum formation follows mitosis. Mol Biol Cell 23(1): 45–58. 22072794 10.1091/mbc.E11-05-0434 PMC3248903

[48] Meitinger F, Petrova B, Lombardi IM, Bertazzi DT, Hub B, et al (2010) Targeted localization of Inn1, Cyk3 and Chs2 by the mitotic-exit network regulates cytokinesis in budding yeast. J Cell Sci 123(11): 1851–1861. 20442249 10.1242/jcs.063891

[49] Janiszewski M, Lopes LR, Carmo AO, Pedro MA, Brandes RP, et al (2005) Regulation of NAD(P)H oxidase by associated protein disulfide isomerase in vascular smooth muscle cells. J Biol Chem 280(49): 40813–40819. 16150729 10.1074/jbc.M509255200

[50] Fernandes DC, Manoel AH, Wosniak J, Jr, Laurindo FR (2009) Protein disulfide isomerase overexpression in vascular smooth muscle cells induces spontaneous preemptive NADPH oxidase activation and Nox1 mRNA expression: Effects of nitrosothiol exposure. Arch Biochem Biophys 484(2): 197–204. 19402212 10.1016/j.abb.2009.01.022

[51] Laurindo FR, Pescatore LA, de Castro Fernandes D (2012) Protein disulfide isomerase in redox cell signaling and homeostasis. Free Radic Biol Med 52(9): 1954–1969. 22401853 10.1016/j.freeradbiomed.2012.02.037

[52] Hatahet F, Ruddock LW (2009) Protein disulfide isomerase: A critical evaluation of its function in disulfide bond formation. Antioxidants & Redox Signaling 11(11): 2807–2850. 19476414 10.1089/ars.2009.2466

[53] Pagani M, Pilati S, Bertoli G, Valsasina B, Sitia R (2001) The C-terminal domain of yeast Ero1p mediates membrane localization and is essential for function. FEBS Lett 508(1): 117–120. 11707280 10.1016/s0014-5793(01)03034-4

[54] Margittai E, Sitia R (2011) Oxidative protein folding in the secretory pathway and redox signaling across compartments and cells. Traffic 12(1): 1–8. 20716108 10.1111/j.1600-0854.2010.01108.x

[55] Brown DI, Griendling KK (2009) Nox proteins in signal transduction. Free Radic Biol Med 47(9): 1239–1253. 19628035 10.1016/j.freeradbiomed.2009.07.023 PMC2763943

[56] Tenberge KB (2004) Morphology and cellular organization in *Botrytis* interaction with plants. In: Elad Y, Williamson B, Tudzynski P, Delen N, editors. *Botrytis*: biology, pathology and control. Dordrecht Boston London: Kluwer Academic Publishers. 67–84.

[57] Jonkers W, van Kan JAL, Tijm P, Lee YW, Tudzynski P, et al (2011) The FRP1 F-box gene has different functions in sexuality, pathogenicity and metabolism in three fungal pathogens. Mol Plant Pathol 12(6): 548–563. 21722294 10.1111/j.1364-3703.2010.00689.x PMC6640539

[58] Buttner P, Koch F, Voigt K, Quidde T, Risch S, et al (1994) Variations in ploidy among isolates of *Botrytis cinerea*: Implications for genetic and molecular analyses. Curr Genet 25(5): 445–450. 8082191 10.1007/BF00351784

[59] Miller (1972) Experiments in molecular genetics. New York: Cold Spring Harbor Laboratory.

[60] Pontecorvo G, Roper JA, Hemmons LM, MacDonald KD, Bufton AWJ (1953) The genetics of *Aspergillus nidulans* . Advances in Genetics Incorporating Molecular Genetic Medicine 5: 141–238. 10.1016/s0065-2660(08)60408-3 13040135

[61] Colot HV, Park G, Turner GE, Ringelberg C, Crew CM, et al (2006) A high-throughput gene knockout procedure for *Neurospora* reveals functions for multiple transcription factors. Proc Natl Acad Sci U S A 103(27): 10352–10357. 16801547 10.1073/pnas.0601456103 PMC1482798

[62] Christianson TW, Sikorski RS, Dante M, Shero JH, Hieter P (1992) Multifunctional yeast high-copy-number shuttle vectors. Gene 110(1): 119–122. 1544568 10.1016/0378-1119(92)90454-w

[63] Winston F, Dollard C, Ricupero-Hovasse SL (1995) Construction of a set of convenient *Saccharomyces cerevisiae* strains that are isogenic to S288C. Yeast 11(1): 53–55. 7762301 10.1002/yea.320110107

[64] Schumacher J (2012) Tools for *Botrytis cinerea*: New expression vectors make the gray mold fungus more accessible to cell biology approaches. Fungal Genet Biol 49(6): 483–497. 22503771 10.1016/j.fgb.2012.03.005

[65] Gronover CS, Kasulke D, Tudzynski P, Tudzynski B (2001) The role of G protein alpha subunits in the infection process of the gray mold fungus *Botrytis cinerea* . Mol Plant Microbe Interact 14(11): 1293–1302. 11763127 10.1094/MPMI.2001.14.11.1293

[66] Cenis JL (1992) Rapid extraction of fungal DNA for PCR amplification. Nucleic Acids Res 20(9): 2380. 1594460 10.1093/nar/20.9.2380 PMC312363

[67] Sambrook J, Fritsch EF and Maniatis T (1989) A laboratory manual, 2nd edn. New York: Cold Spring Harbor Laboratory.

[68] Vandervlugtbergmans CJB, Brandwagt BF, Vantklooster JW, Wagemakers CAM, van Kan JAL (1993) Genetic-variation and segregation of DNA polymorphisms in *Botrytis cinerea* . Mycol Res 97: 1193–1200.

[69] Doehlemann G, Berndt P, Hahn M (2006) Different signalling pathways involving a G-alpha protein, cAMP and a MAP kinase control germination of *Botrytis cinerea* conidia. Mol Microbiol 59(3): 821–835. 16420354 10.1111/j.1365-2958.2005.04991.x

[70] Vogel H (1956) A convenient growth medium. Microbial Genetics Bulletin 13: 42–46.

[71] Klimpel A, Gronover CS, Williamson B, Stewart JA, Tudzynski B (2002) The adenylate cyclase (BAC) in *Botrytis cinerea* is required for full pathogenicity. Molecular Plant Pathology 3(6): 439–450. 20569351 10.1046/j.1364-3703.2002.00137.x

[72] Araki K, Nagata K (2011) Functional in vitro analysis of the ERO1 protein and protein-disulfide isomerase pathway. J Biol Chem 286(37): 32705–32712. 21757736 10.1074/jbc.M111.227181 PMC3173198

