# Correction: The NADPH Oxidase Complexes in *Botrytis cinerea*: Evidence for a Close Association with the ER and the Tetraspanin Pls1

**DOI:** 10.1371/annotation/84c258cf-4f98-43d8-a4f0-7032cba36f22

**Published:** 2013-06-18

**Authors:** Ulrike Siegmund, Jens Heller, Jan A. L. van Kan, Paul Tudzynski

The third author's name was spelled incorrectly. The correct name is: Jan A. L. van Kan. The correct citation is: Siegmund U, Heller J, van Kan JAL, Tudzynski P (2013) The NADPH Oxidase Complexes in Botrytis cinerea: Evidence for a Close Association with the ER and the Tetraspanin Pls1. PLoS ONE 8(2): e55879. doi:10.1371/journal.pone.0055879

