# Supplementary figures and images for: The NADPH Oxidase Complexes in Botrytis cinerea: Evidence for a Close Association with the ER and the Tetraspanin Pls1
Source: PLoS One. 2013 Feb 13;8(2):e55879. doi: 10.1371/journal.pone.0055879 (PMC3572182; doi:10.1371/journal.pone.0055879)

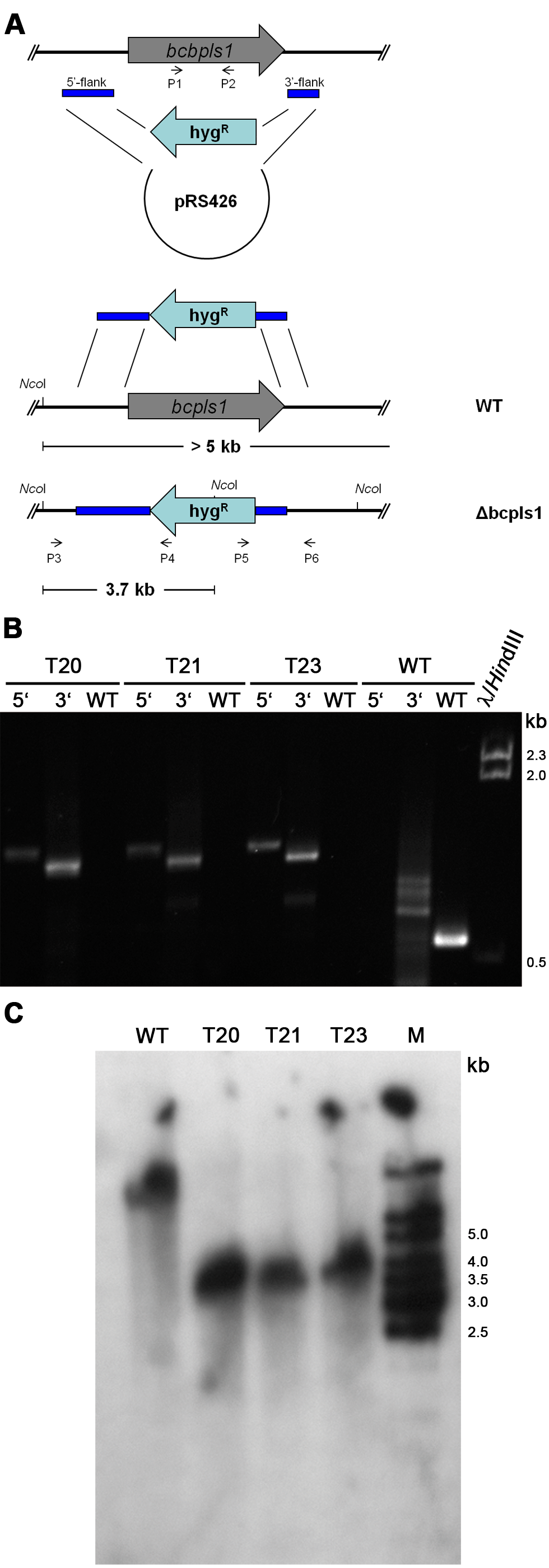

Supplement: Figure S1 — Deletion of the gene bcpls1. A: Schematic overview of the deletion strategy. For the detection of homologous integration of the replacement fragments primers were used that bind upstream the 5′-region and within the terminator of the resistance cassette (P3/P4; 5′), or in the promoter of the resistance cassette and downstream the 3′-prime region (P5/P6; 3′), respectively. For detection of purified mutants, primers binding within the wild-type bcpls1 were used (P1/P2; WT). For Southern blot analyses genomic DNA was digested using the restriction enzyme NcoI that cuts within the resistance cassette but not within bcpls1. Using the 5′ flank as a probe, in the wild-type a fragment larger than 5 kb was expected and in the mutant a fragment of 3.7 kb. B: Diagnostic PCR showing homologous integration of the knock-out fragment at bcpls1. C: Southern blot showing no further integrations of the knock-out fragment. (TIF) [file pone.0055879.s001.tif]

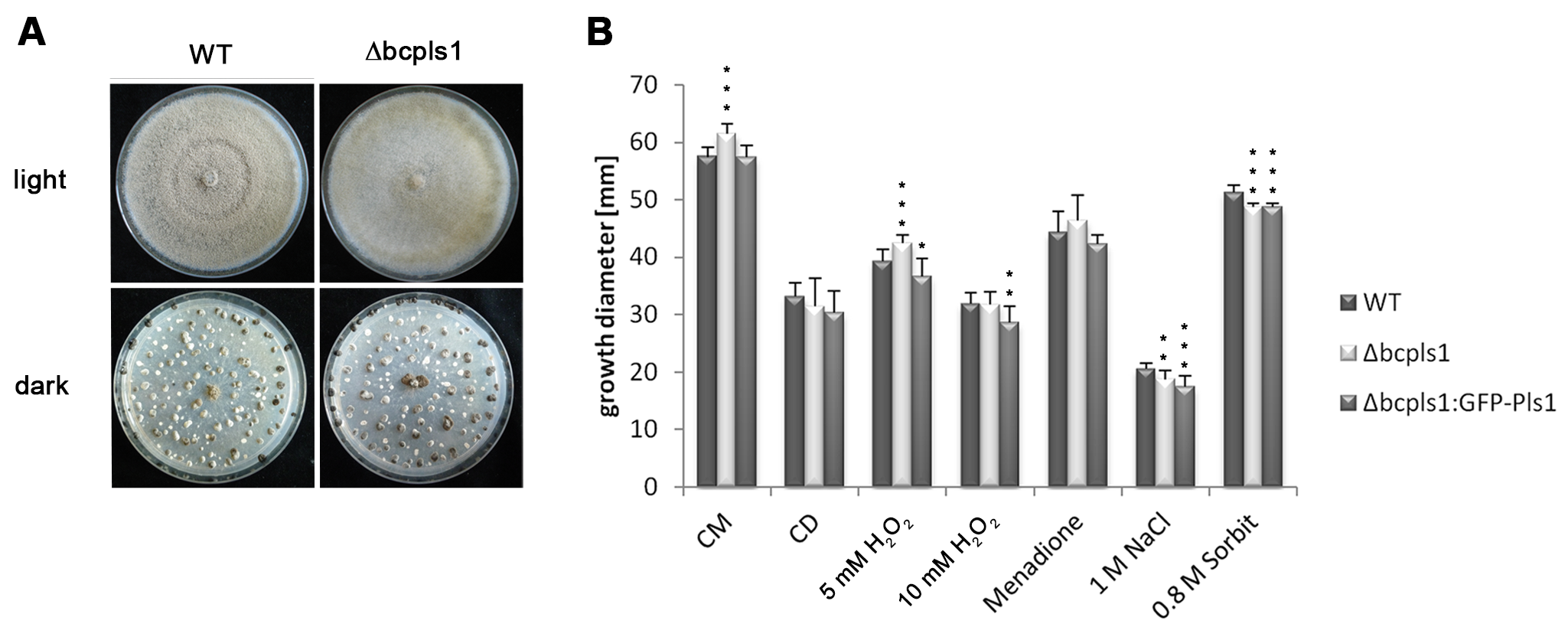

Supplement: Figure S2 — Comparison of growth and differentiation of B. cinerea B05.10 WT and Δbcpls1. A: For analysis of light-dependent differentiation, strains were cultivated on CM for 7 days with a rhythm of 12 h light and 12 h darkness (light) or for 3 weeks in complete darkness (dark). Δbcpls1 shows no differences to the WT. B: Plate assays showing growth rates of B. cinerea B05.10 and Δbcpls1 on stress causing media. As a control CM was used, oxidative stress was induced using 5 mM H2O2, 10 mM H2O2 and 500 mM menadione, osmotic stress was induced using 1 M NaCl and 1 M sorbitol. Colony diameters were measured 3 days after the inoculation. The indicated values are means of five different plates; standard deviations are indicated by the error bars. Asterisks above the bars denote significant differences in the measurements of the indicated strains to the WT. * = P<0.05; ** = P<0.01; *** = P<0.001. (TIF) [file pone.0055879.s002.tif]

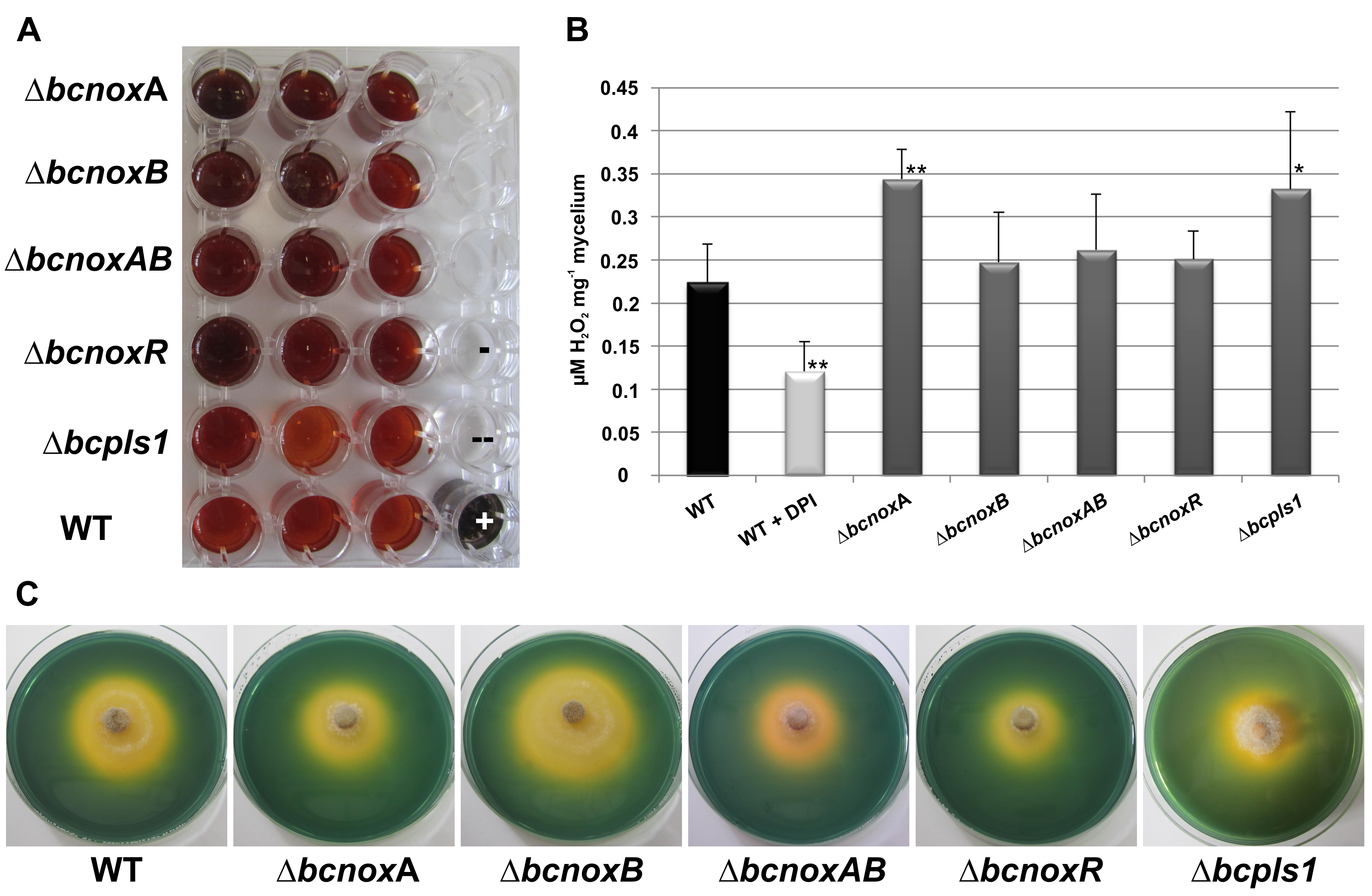

Supplement: Figure S3 — Production of H2O2 and oxalic acid in Δbcpls1, ΔbcnoxA, ΔbcnoxB, ΔbcnoxAB, ΔbcnoxR and the WT. A: Production of H2O2 was monitored using DAB staining. 25 mg of fresh mycelia were placed in each well to exclude staining differences evolving from differing growth rates of the strains. Strains were previously grown on CM overlayed with cellophane for 3 days. The mycelium was inoculated with DAB solution for 1.5 h in the dark. As negative controls, one well was only filled with DAB solution (-) and one with DAB solution and 1 µl H2O2 (–). DAB solution, 1 µl H2O2 and 1 µl horseradish peroxidase served as a positive control (+). B: Quantitative analysis of H2O2 production using Amplex Red Peroxide Assay. 20 mg of fresh mycelia were incubated in the Amplex Red working solution. Strains were previously grown on complete medium (CM) overlayed with cellophane for 3 days, for inhibition of flavoenzymes 100 µM DPI were added to the medium. Fluorescence emission was detected after 30 min at 590 nm (excitation 560 nm). C: Acidification of growth medium by oxalic acid production was monitored using the pH indicator bromothymol blue, which was added to the media and turns yellow with decreasing pH value. Plates were inoculated with young mycelium and grown for 3 days. Asterisks above the bars denote significant differences in the measurements of the indicated strains to the WT. * = P<0.05; ** = P<0.01. (TIF) [file pone.0055879.s003.tif]

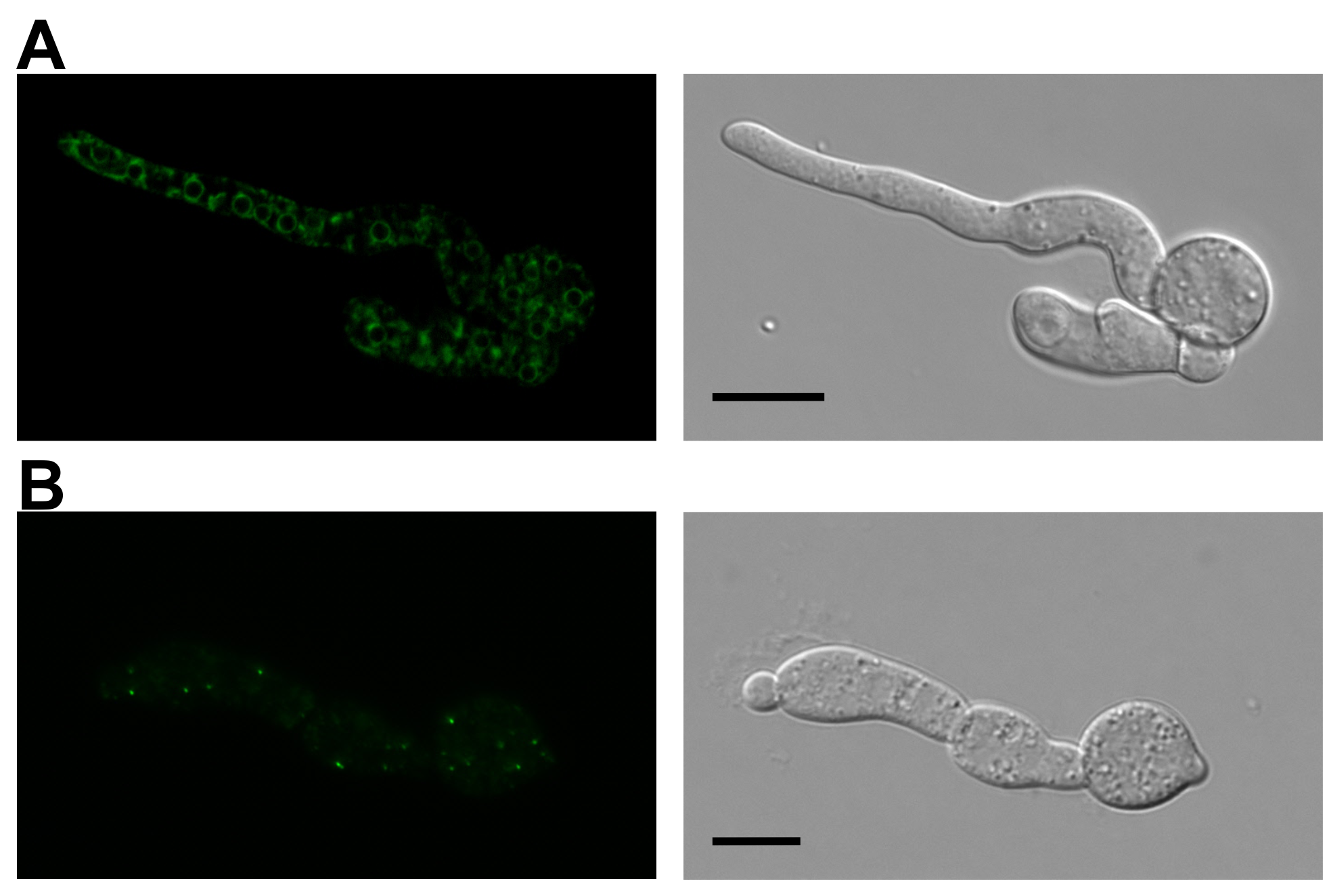

Supplement: Figure S4 — Cellular localization of BcNoxA and BcNoxR in the respective deletion mutants. Protein localization was determined by epifluorescence microscopy in germlings (in Gamborgs B5+2% glucose) of the strains ΔbcnoxA:GFP-NoxA and ΔbcnoxR:GFP-NoxR expressing gfp-bcnoxA and gfp-noxR gene fusions, respectively. A: BcNoxA localized to intracellular membrane structures and at times also to the plasma membrane. B: BcNoxR accumulated in cellular granules, which were distributed irregularly all over the hyphae. Scale bars = 10 µm. (TIF) [file pone.0055879.s004.tif]

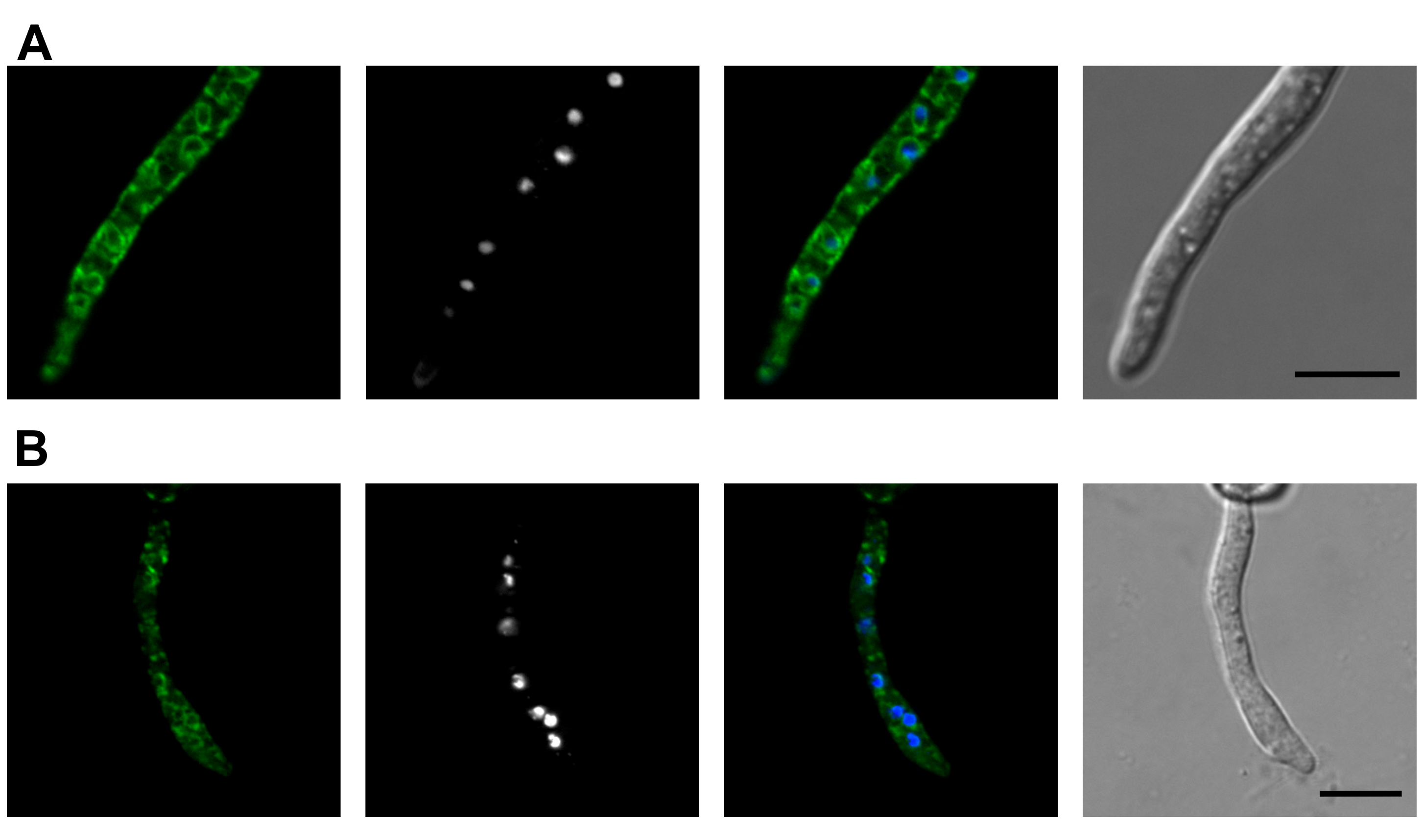

Supplement: Figure S5 — Localization of BcNoxA and BcNoxB compared with Hoechst-staining of the nuclei. Protein localization was determined by epifluorescence microscopy in germlings of the strains WT:GFP-NoxA and WT:NoxB-GFP expressing gfp-bcnoxA and bcnoxB-gfp gene fusions, respectively. 10-µl droplets of a conidial suspension in GB5 medium (105 conida/ml) were placed on a glass slide and allowed to germinate over night before microscopic analyses were performed. A: BcNoxA localized to intracellular membrane structures and at times also to the plasma membrane. Hoechst staining and a respective overlay show that the intracellular structures surround the nuclei (from left to right: GFP-NoxA, Hoechst, overlay, white light). B: BcNoxB localized to similar intracellular membrane structures and to the plasma membrane as visible for BcNoxA. Hoechst staining and a respective overlay show that the intracellular structures surround the nuclei (from left to right: NoxB-GFP, Hoechst, overlay, white light). Scale bars = 10 µm. (TIF) [file pone.0055879.s005.tif]
