# Supplementary material for: The NADPH Oxidase Complexes in Botrytis cinerea: Evidence for a Close Association with the ER and the Tetraspanin Pls1
Source: PLoS One. 2013 Feb 13;8(2):e55879. doi: 10.1371/journal.pone.0055879 (PMC3572182; doi:10.1371/journal.pone.0055879)
Supplement: Table S1 — Predicted localization of Nox proteins from various organisms. Protein sequences of NoxA/1 and NoxB/2 from B. cinerea, A. nidulans, M. oryzae, S. sclerotiorum, E. festucae, P. anserina, N. crassa and Trichoderma reesei as well as Nox1, Nox2 and Nox4 from Homo sapiens were used to predict their cellular localization using ProtComp v. 9.0 (http://linux1.softberry.com/berry.phtml?topic=protcompan&group=programs&subgroup=proloc). (DOCX) [file pone.0055879.s006.docx]

|  | **predicted cellular localization** | | |
| --- | --- | --- | --- |
| **Organism** | **NoxA/1** | **NoxB/2** | **Nox4** |
| *B. cinerea* | PM | PM | - |
| *A. nidulans* | PM | PM | - |
| *S. sclerotiorum* | PM | PM | - |
| *M. oryzae* | PM | PM | - |
| *E. festucae* | PM | PM | - |
| *T. reseei* | PM | ER | - |
| *P. anserina* | PM | PM | - |
| *N. crassa* | PM | PM | - |
| *H. sapiens* | PM | PM | PM |
