# Supplementary material for: The NADPH Oxidase Complexes in Botrytis cinerea: Evidence for a Close Association with the ER and the Tetraspanin Pls1
Source: PLoS One. 2013 Feb 13;8(2):e55879. doi: 10.1371/journal.pone.0055879 (PMC3572182; doi:10.1371/journal.pone.0055879)
Supplement: Table S2 — Oligonucleotide primers used in this study. (DOCX) [file pone.0055879.s007.docx]

| **No.** | **Name** | **Sequence (5'-3')** | **purpose** |
| --- | --- | --- | --- |
| 1 | Pls1_forw | GGTTCGCCTTGACGACGAAGACTGG | diaPCR |
| 2 | Pls1_rev | CGAAGAAGCCATGAAGTTTGAGCC | diaPCR |
| 3 | Dia_Pls1_forw | CCGAATTTGAAGTCTGCGTCTACCC | diaPCR |
| 4 | pCSN44-hph-trpC-T | GGAATAGAGTAGATGCCGACCGG | diaPCR |
| 5 | Dia_Pls1_rev | CCCTCTTCCCATCTCCACAACCCCC | diaPCR |
| 6 | pCSN44-trpC-P | CCTCCACTAGCTCCAGCCAAGCCC | diaPCR |
| 7 | Pls1_5f | GTAACGCCAGGGTTTTCCCAGTCACGACGCAAGCTTCCTGAACCTTTCTACCG | deletion |
| 8 | Pls1_5r | ATCCACTTAACGTTACTGAAATCTCCAACGTTGTCTTAGTAAGAGGACGTCCG | deletion |
| 9 | Pls1_3f | CTCCTTCAATATCATCTTCTGTCTCCGACGATGGAAATGGGTGGCTAGATG | deletion |
| 10 | Pls1_3r | GCGGATAACAATTTCACACAGGAAACAGCCATCTCCCATATCCTACACCACGC | deletion |
| 11 | hphF-trpC-P | GTCGGAGACAGAAGATGATATTGAAGGAGC | deletion |
| 12 | hphR-trpC-T | GTTGGAGATTTCAGTAACGTTAAGTGGAT | deletion |
| 13 | TrpC-SpeI-F1 | ACTAGTGATATTGAAGGAGCATTTTTTGGGC | gfp |
| 14 | TrpC-nat1-R2 | AATTCCAGGCCTGATGCTTTGGTTTAGGGTTAGGCCCC | gfp |
| 15 | noxR-gfp_Ye_f | GGGAATGGATGAACTTTACAAAATGTCTTTGAAACAGGTGAGTGG | gfp |
| 16 | noxR-gfp_Ye_r | CATACATCTTATCTACATACGATCACGCTTTTACTACCCAAATC | gfp |
| 17 | bcnoxB_PoliC_F | CTCCATCACATCACAATCGATCCAACCATGTCTGAAAAGGCTTATGACAG | gfp |
| 18 | bcnoxB_OGFP_R | CTTACCTCACCCTTGGAAACCATGAAATTTTCTTTGCCCCAGCAG | gfp |
| 19 | bcnoxA_OGFP_F | GGGAATGGATGAACTTTACAAAATGGGTGCTGTACAGTTTTTG | gfp |
| 20 | bcnoxA_Tgluc_R | CATACATCTTATCTACATACGCTAGAAATGTTCTTTCCAAAACG | gfp |
